# The scorpion peptide Eval418 inhibits duck Tembusu virus replication by disrupting viral structure and early-stage infection

**DOI:** 10.3389/fvets.2026.1944118

**Published:** 2026-09-21

**Authors:** Yuting Cheng, Qi Feng, Pei Zhang, Chunyan Ma, Wenjing Xie, Anping Wang, Zhi Wu, Wenfeng Jia, Qingguo Wu, Lixia Miao, Shanyuan Zhu

**Affiliations:** 1 Jiangsu Key Laboratory of Veterinary Bio-Pharmaceutical High Technology Research, Engineering Technology Research Center for Modern Animal Science and Novel Veterinary Pharmaceutic Development, Jiangsu Agri-Animal Husbandry Vocational College, Taizhou, China; 2 Institutional Center for Shared Technologies and Facilities of Wuhan Institute of Virology, Chinese Academy of Sciences, Wuhan, China; 3 Department of Biochemistry, School of Basic Medical Sciences, Wuhan University, Wuhan, China

**Keywords:** antiviral activity, Duck Tembusu virus (DTMUV), Eval418, molecular docking, scorpion peptide, viral replication

## Abstract

**Introduction:**

Duck Tembusu virus (DTMUV) causes egg-drop syndrome and neurological symptoms in ducks, resulting in significant economic losses. Currently, no specific antiviral drugs are available for DTMUV infection. Scorpion-derived peptides have emerged as promising lead compounds for antiviral drug development. In this study, we investigated the antiviral activity and mechanism of the scorpion peptide Eval418 against DTMUV infection *in vitro*.

**Methods:**

DF-1 cells were infected with DTMUV at an MOI of 0.1 and treated with Eval418 (0–20 μM), with Ribavirin as a positive control. Antiviral activity was assessed by Western blot, RT-qPCR, and immunofluorescence assays targeting the DTMUV NS3 protein. Cytotoxicity and hemolytic activity were evaluated in DF-1, DEF, chicken and duck red blood cells the influence of duck serum on Eval418 was examined. Time-of-addition, free virion, attachment, and entry/fusion assays were performed, along with transmission electron microscopy and molecular docking/MD simulations of the NS3-Eval418 complex.

**Results:**

Eval418 inhibited DTMUV replication in a concentration-dependent manner, achieving over 99% inhibition at 10 μM, with no significant cytotoxicity or hemolytic activity up to 120 μM in all tested cells. Mechanistic studies revealed that Eval418 acted primarily during the coaddition stage by directly inactivating free virions, blocking viral attachment, and inhibiting viral entry/fusion. TEM confirmed that Eval418 directly disrupted the morphological integrity of DTMUV virions, causing structural collapse. MD simulations showed that Eval418 binds stably to the NS3 binding pocket. Eval418 also inhibited two genetically distinct DTMUV isolates, whereas prolonged incubation with duck serum progressively reduced its antiviral activity.

**Conclusion:**

These findings establish Eval418 as a promising lead compound for the development of antiviral therapeutics against DTMUV infection as a model flavivirus. Its potential efficacy against other flaviviruses requires further investigation.

## 1 Introduction

Duck Tembusu virus (DTMUV), a single-stranded positive-sense RNA virus belonging to the genus *Flavivirus* within the family *Flaviviridae*, was first identified in China in 2010 and has since been confirmed as the causative agent of duck egg-drop syndrome (DEDS) (1, 2). Infection with DTMUV can lead to a sharp decline in egg production and hemorrhagic ovarian lesions in laying ducks, as well as neurological symptoms and even mortality in meat ducks, resulting in significant economic losses to the duck industry (3). Characterized by a high mutation rate and a broad host range, DTMUV not only infects waterfowl such as ducks and geese but also poultry like chickens and sparrows, as well as mammals, raising concerns regarding potential cross-species transmission that complicates its control (4–6). Current prevention and control strategies against DTMUV primarily rely on live attenuated vaccines, inactivated vaccines, and DNA vaccines (7–12). However, the emergence of viral variants can reduce vaccine efficacy, and the lack of specific antiviral drugs highlights the urgent need for the development of novel control strategies (13, 14).

Scorpions are ancient terrestrial organisms that have inhabited the Earth for over 400 million years. To prey and survive, they have evolved a unique venom system containing neurotoxins, antiviral peptides (AVPs), enzymes, and other components. Natural AVPs derived from scorpion venom and their derivative peptides have demonstrated promising antiviral activities, some of which have been investigated in previous reports. For instance, the recombinant scorpine (RScp) can inhibit Dengue-2 virus replication in C6/36 cells (15). The natural amphipathic α-helical peptide Hp1090 can directly interact with the HCV envelope and reduce viral infectivity (16). Scorpion defensin BmKDfsin3 inhibits HCV replication, which is associated with the modulation of p38 MAPK activation (17). These studies indicate that scorpion-derived peptides, owing to their unique mechanisms of action and low cytotoxicity, have emerged as promising lead compounds in antiviral drug development.

In our previous research, a peptide designated Eval418 with activity against herpes simplex virus type 1 (HSV-1) was identified from a scorpion venom peptide library. Based on its sequence and antiviral characteristics, a histidine-rich derivative of Eval418 was designed, which demonstrated significantly enhanced potential for further development as an anti-HSV-1 agent (18). Eval418 is a scorpion venom-derived peptide from the venom gland of *E. validus*, consisting of 13 amino acid residues. However, whether Eval418 possesses antiviral activity against viruses of the family *Flaviviridae*, particularly DTMUV, and its underlying mechanism remain unclear.

Therefore, this study utilized DTMUV from the *Flaviviridae* family as a model to investigate the inhibitory effect of Eval418 on DTMUV replication *in vitro* and to systematically elucidate its stage of action and molecular mechanism. The findings will provide both a new candidate molecule and a theoretical foundation for the development of natural peptide-based antiviral drugs against DTMUV.

## 2 Materials and methods

### 2.1 Cells, virus, and scorpion peptide

Chicken embryo fibroblast (DF-1) cells and Duck embryo fibroblasts‌ (DEF) were maintained in our laboratory and cultured in DMEM medium supplemented with 10% fetal bovine serum, 100 U/mL penicillin, and 100 μg/mL streptomycin. The cells were incubated at 37 °C in a humidified incubator with 5% CO₂.

The DTMUV strain-2015 (DTMUV-2015) and DTMUV strain-201909 (DTMUV) used in this study were isolated and maintained by our laboratory. The DTMUV-2015 strain was isolated from a duck farm in Xuzhou, Jiangsu Province, China, in May 2015, and passaged in SPF duck embryos (CCTCC No. V202261). The DTMUV strain (DTMUV-201909) was isolated from a duck farm in Zhenjiang, Jiangsu Province, China, in September 2019, and passaged in SPF duck embryos (CCTCC No. V202262). The viral titer was determined using a ten-fold dilution method, where diluted virus was inoculated onto a 96-well cell culture plate, and the TCID_50_ was calculated according to the Reed-Muench method.

The scorpion peptide Eval418 was kindly provided by Professor Zhijian Cao from the Hubei University of Technology. It was dissolved in dimethyl sulfoxide (DMSO) prior to use and stored at −80 °C. The final concentration of DMSO in all cell culture experiments was maintained at ≤0.1% (v/v), and the corresponding concentration of DMSO was included as a vehicle control in all assays.

### 2.2 Antibodies and reagents

Mouse-derived polyclonal antibodies against DTMUV NS3 (320–619 aa) were prepared in our laboratory. The following commercial reagents were employed: ChamQ Universal SYBR qPCR Master Mix (Vazyme, Q711-02), HiScript® III RT SuperMix for qPCR (+gDNA wiper) (Vazyme, R323-01), FastPure Cell/Tissue Total RNA Isolation Kit V2 (Vazyme, RC112-01), ProteinFind® Anti-β-Actin Mouse Monoclonal Antibody (TransGen, HC201-02), Alexa Fluor 488-conjugated goat anti-mouse IgG (Yeasen, 33206ES60), HRP-conjugated goat anti-mouse IgG (Yeasen, 33201ES), and an MTT assay kit (Solarbio, M1020).

### 2.3 Physicochemical property analysis

The physicochemical properties of Eval418, including molecular weight, isoelectric point (pI), estimated solubility, and half-life, were predicted using online bioinformatics tools: ProtParam^1^ for basic physicochemical parameters. The Hopp & Woods hydrophilicity profile was generated using PepCalc^2^. Helical wheel projection was generated using NetWheels^3^. Tertiary structure prediction was performed using AlphaFold2^4^.

### 2.4 MTT assay

To analyze the cytotoxic effect of Eval418 on DF-1 and DEF cells, cytotoxicity was assessed using an MTT assay as described in our laboratory’s previous publication (17). Briefly, DF-1 and DEF cells were seeded into 96-well plates (10^4^ cells per well) and incubated for 24 h at 37 °C. The culture medium was then replaced with fresh medium containing various concentrations of Eval418, and the cells were incubated for an additional 48 h at 37 °C. Subsequently, the medium was removed, and 10 μL of MTT solution mixed with 90 μL of medium was added to each well, followed by incubation for 4 h at 37 °C. The medium was then replaced with 110 μL of Formazan solubilization solution, and the plate was gently shaken at room temperature for 10 min. The absorbance was measured at a wavelength of 490 nm using a microplate reader.

### 2.5 Hemolysis assay

Fresh anticoagulated chicken and duck whole blood was collected from healthy chickens and ducks by venipuncture and immediately processed for the hemolysis assay. The blood was diluted with an appropriate volume of PBS solution, and gently mixed. It was then centrifuged at 2,000 rpm for 5 min. The supernatant and the white buffy coat layer were carefully aspirated and discarded. The pelleted red blood cells (RBCs) were resuspended in PBS solution and centrifuged again for washing. This washing step was repeated three times until the supernatant became clear and transparent. Finally, the washed RBCs were resuspended in PBS to prepare a 2% (v/v) suspension for later use. In a 96-well plate, the following groups were set up, each with three replicate wells: negative control (PBS + RBC suspension), positive control (1% Triton X-100 + RBC suspension), experimental group (peptide sample + RBC suspension), and sample blank (peptide sample + PBS). The contents of each well were gently mixed, and the plate was incubated at 37 °C for 1 h. After incubation, 100 μL of the supernatant from each well was transferred to a new 96-well plate. The optical density (OD) of each supernatant was measured at a wavelength of 540 nm using a microplate reader. The hemolysis rate (%) was calculated as follows: [(OD_experimental_ – OD_negative control_)/(OD_positive control_ – OD_negative control_)] × 100%, where OD_experimental_ represents the corrected OD value of the experimental group after subtraction of the corresponding “sample blank” value.

### 2.6 Effect of duck serum incubation on the antiviral activity of Eval418

Duck serum used in this study was obtained from residual samples previously collected from healthy ducks under an approved animal ethics protocol. Prior to use, the serum was heat-inactivated at 56 °C for 30 min to inactivate complement and filtered through a 0.22 μm filter. To evaluate the effect of duck serum on the antiviral activity of Eval418, three experimental groups were set up: (1) 45 μL heat-inactivated duck serum + 5 μL of 10 mM Eval418 stock solution; (2) 45 μL DMEM medium + 5 μL of 10 mM Eval418 stock solution (serum-free control); and (3) 45 μL heat-inactivated duck serum + 5 μL DMSO (serum solvent control). All mixtures were incubated at 37 °C for 0, 2, 4, 8, 12, or 24 h. After the designated incubation periods, 10 μL of each incubation mixture was taken and added to the virus inoculum (final volume 500 μL), resulting in a 50-fold dilution of the incubation mixture. The final concentrations in the cell culture system were: Eval418 at 20 μM, DMSO at 0.02%, and duck serum at 1.8% (v/v). The mixture was then added to DF-1 cells for viral adsorption. Following viral adsorption, cells were washed and cultured in fresh medium. At the indicated time points, cells were collected for Western blot analysis of DTMUV NS3 protein expression.

### 2.7 Eval418 treatment and DTMUV infection *in vitro*

To investigate the antiviral effect of Eval418 against DTMUV, DF-1 cells were seeded in 12-well plates at a density of 2 × 10^5^ cells per well to reach approximately 70% confluency. The following day, cells were pre-incubated with Eval418 at 37 °C for 2 h to allow peptide-cell interaction. The supernatant was then removed, and cells were infected with DTMUV at an MOI of 0.1 in the presence of fresh medium containing the same concentration of Eval418 (0, 2.5, 5, 10, and 20 μM) or Ribavirin (3 μM). After 4 h, the inoculum was replaced with fresh DMEM containing 10% FBS and the same concentration of Eval418 (0, 2.5, 5, 10, and 20 μM) or Ribavirin (3 μM), and the cells were incubated for an additional 48 h. This three-step design (pre-incubation, co-infection, and post-infection continuous treatment) simulates both pre-exposure prophylaxis and post-exposure continuous therapy. To evaluate the antiviral activity of Eval418 against genetically distinct DTMUV isolates, identical experiments were performed using the DTMUV-2015 isolate under the same experimental conditions. Subsequently, cell culture supernatants were collected for viral titer determination, and cell pellets were harvested for immunofluorescence or Western blot analysis of DTMUV NS3 protein expression. Reverse transcription-polymerase chain reaction (RT-qPCR) was performed to detect DTMUV RNA expression.

### 2.8 Phylogenetic analysis

Representative DTMUV genome sequences were retrieved from the National Center for Biotechnology Information (NCBI) database. Phylogenetic analysis was performed using the isolate used in this study together with representative DTMUV isolates collected during different epidemic periods. Multiple sequence alignment was performed using ClustalW, and a phylogenetic tree was constructed using the Neighbor-Joining method implemented in MEGA 11 with 1,000 bootstrap replicates. Sitiawan virus was included as the outgroup.

### 2.9 Pretreatment, coaddition, and postinfection assays

To determine the specific stage at which Eval418 inhibits DTMUV replication, DF-1 cells were infected with DTMUV (MOI = 1) under three different treatment conditions, following procedures adapted from previous studies (17): Pretreatment: DF-1 cells were pretreated with Eval418 (10 μM) or Ribavirin (3 μM) for 2 h prior to DTMUV infection. After viral adsorption for 4 h, cells were washed twice with PBS and cultured in fresh medium for 48 h before sample collection. Coaddition: Eval418 (10 μM) or Ribavirin (3 μM) was simultaneously mixed with DTMUV during viral adsorption for 4 h. Cells were washed twice with PBS and further cultured for 48 h before analysis. Postinfection: DF-1 cells were first infected with DTMUV for 4 h. After removal of the inoculum and washing with PBS, fresh medium containing Eval418 (10 μM) or Ribavirin (3 μM) was added, followed by incubation for 48 h before sample collection. Viral RNA levels were determined by RT-qPCR and viral protein expression was analyzed by Western blotting.

### 2.10 Effect of Eval418 on free virion, viral attachment, and entry/fusion

To further elucidate which specific early stage of the DTMUV life cycle is inhibited by Eval418, DF-1 cells were infected with DTMUV (MOI = 1) under three different treatment conditions, based on procedures adapted from previous studies (17):

Effect on free virion: DTMUV was mixed with 10 μM Eval418 and incubated at 37 °C for 2 h. This mixture was then used to infect DF-1 cells for 4 h. Subsequently, the inoculum was replaced with fresh DMEM containing 10% FBS, and the cells were incubated at 37 °C for 48 h. Cell pellets were collected for Western blot analysis of DTMUV NS3 protein expression and RT-qPCR to detect DTMUV RNA levels, to evaluate the virucidal activity of Eval418.

Effect on viral attachment: A mixture of DTMUV and 10 μM Eval418 was added to DF-1 cells, followed by incubation at 4 °C for 4 h to allow viral binding while blocking internalization. The inoculum was then replaced with fresh DMEM containing 10% FBS, and the cells were incubated at 37 °C for 48 h. Cell pellets were collected for Western blot analysis of DTMUV NS3 protein expression and RT-qPCR to detect DTMUV RNA levels, to assess the effect of Eval418 on viral attachment. Additionally, to directly evaluate the effect of Eval418 on viral binding, a virus attachment assay was performed at 4 °C. DF-1 cells were incubated with DTMUV (MOI = 1) in the presence or absence of 10 μM Eval418 at 4 °C for 4 h to allow viral attachment while minimizing internalization. After incubation, cells were thoroughly washed three times with pre-chilled PBS to remove unbound virus. For direct binding assessment, total cellular RNA was immediately extracted, and cell-associated DTMUV RNA levels were quantified by RT-qPCR.

Effect on viral entry/fusion: DF-1 cells were incubated with DTMUV at 4 °C for 4 h to allow viral attachment. The unbound virus was removed, and the cells were incubated at 37 °C for 2 h in fresh DMEM containing 10% FBS and 10 μM Eval418 to facilitate viral entry/fusion in the presence of the peptide. Finally, the medium was replaced with fresh DMEM containing 10% FBS, and the cells were incubated at 37 °C for 48 h. Cell pellets were collected for Western blot analysis of DTMUV NS3 protein expression and RT-qPCR to detect DTMUV RNA levels, to evaluate the effect of Eval418 on viral entry/fusion.

### 2.11 Indirect immunofluorescence assay

The cells to be examined were fixed with ice-cold methanol for 15 min and then blocked with 5% BSA for 1 h. Subsequently, the cells were incubated with mouse anti-DTMUV NS3 polyclonal antibody (1:500) at 4 °C overnight. After washing, Alexa Fluor 488-conjugated goat anti-mouse secondary antibody (1:250) was added and incubated at room temperature for 2 h in the dark. Finally, the nuclei were stained with DAPI at room temperature, and the samples were observed under a fluorescence microscope. Alexa Fluor 488 was excited at a wavelength of 488 nm, and DAPI was excited at 408 nm.

### 2.12 RT-qPCR

Cells were collected, and total intracellular RNA was extracted and reverse transcribed to obtain cDNA, which served as the template for the RT-qPCR reaction. The PCR reaction mixture (20 μL) consisted of: 10 μL of 2 × ChamQ SYBR qPCR Master Mix (Low ROX Premixed), 1 μL each of forward and reverse primers (10 μM), and 7 μL of ddH₂O. The PCR cycling protocol was as follows: pre-denaturation at 95 °C for 3 min; followed by 30 cycles of denaturation at 95 °C for 30 s, annealing at 58 °C for 30 s, and extension at 72 °C for 20 s. Each sample was analyzed in triplicate. The relative expression level of the target gene was calculated using the 2^(−ΔΔCt)^ method. The primers for DTMUV were: 5′-GAGGACGATTGATGGGGTCC-3′ (forward) and 5′-CCGTCGAGCATCTGTCCAAT-3′ (reverse). The primers for GAPDH were: 5′- ACTTCAACGGTGACAGCCAT-3′ (forward) and 5′- ACCATCAAGTCCACCACACG-3′ (reverse).

### 2.13 Western blot

Cell pellets were collected and lysed with RIPA lysis buffer on ice for 30 min. The lysates were centrifuged at 12,000 rpm at 4 °C for 15 min. The supernatant was collected, and the protein concentration was determined using the BCA method. For SDS-PAGE, 30 μg of cellular protein per sample was loaded into each well. After electrophoresis, the proteins were transferred onto a polyvinylidene difluoride (PVDF) membrane. The membrane was blocked with 5% skim milk powder at room temperature for 2 h, followed by incubation with mouse anti-DTMUV NS3 polyclonal antibody (1:1,500) at 4 °C overnight. Subsequently, the membrane was incubated with an HRP-conjugated secondary antibody of the corresponding species (1:10,000) at room temperature for 2 h. The target protein bands were visualized and analyzed using a chemiluminescence imaging system.

### 2.14 Transmission electron microscopy (TEM)

DTMUV virions were co-incubated with either PBS or 20 μM Eval418 peptide at 37 °C for 2 h. Subsequently, the incubated viral samples were applied onto 200-mesh var/carbon-coated copper grids for adsorption. After gentle rinsing with deionized water, the samples were negatively stained using a 2% phosphotungstic acid solution (pH 7.0, adjusted with NaOH). The stained grids were then air-dried overnight at room temperature. Finally, different areas of the grids were examined and images were acquired using a 200 kV transmission electron microscope (FEI Tecnai G2 20 TWIN).

### 2.15 Molecular docking and molecular dynamics’ simulation

The complex structure of NS3 and Eval418 was predicted via protein–protein docking using the AlphaFold 3 online server^5^ with default parameters, and the three-dimensional (3D) structure, binding interface, and key residues were analyzed using PyMOL 2.6.1. Subsequently, molecular dynamics simulations of this complex were conducted using GROMACS 2022.3. The parameters and topologies of the complex were generated using the amber99sb-ildn force field. The protein was placed in a cubic box with a minimum distance of 1.0 nm from the box edges, and the box was filled with TIP3P water molecules, with Na^+^ and Cl^−^ ions added to neutralize the system. The entire system underwent energy minimization via the steepest descent method to eliminate non-ideal contacts and atomic overlaps. The system was then subjected to NVT and NPT ensemble simulations, each lasting 100 ps, at 300 K and 1 bar, to ensure thorough pre-equilibration, followed by a 100 ns production MD simulation under periodic boundary conditions with an integration step of 2 fs. The temperature was maintained at 300 K using the V-rescale thermostat, and the pressure at 1 bar using the Parrinello-Rahman barostat. Long-range electrostatic interactions were computed using the Particle Mesh Ewald (PME) method with a Fourier grid spacing of 0.16 nm, and bond lengths were constrained using the LINCS algorithm. The simulation trajectories were visualized using PyMOL 2.6.1, and the binding free energies of the complex were calculated using gmx_MMPBSA^6^.

### 2.16 Ethics statement

All animal procedures were conducted in accordance with the institutional guidelines for the care and use of laboratory animals and were approved by the Animal Ethics Committee of Jiangsu Agri-Animal Husbandry Vocational College (Approval No. Jsahvc-2025-38). Fresh chicken and duck blood used for the hemolysis assay was collected from healthy chickens and ducks by venipuncture using minimally invasive procedures. Duck serum used in this study was obtained from residual samples previously collected under the same approved animal ethics protocol. No animals were euthanized for the purpose of this study.

### 2.17 Statistical analysis

Statistical analysis was performed using Adobe Photoshop CS6 and GraphPad Prism 9. Data are presented as the mean ± SD from at least three independent experiments. Statistical significance was determined using Student’s *t*-test, with *p*-values less than 0.05 considered significant (*, *p* < 0.05; **, *p* < 0.01; ***, *p* < 0.001).

## 3 Results

### 3.1 Physicochemical property analysis of the scorpion peptide Eval418

A systematic analysis of the physicochemical properties of Eval418 was conducted. This peptide consists of 13 amino acid residues with a molecular weight of 1528.81 Da. The theoretical isoelectric point (pI) is 6.85, which is close to neutral. Sequence-based predictions indicated poor water solubility for this peptide. The estimated half-life in an *in vitro* mammalian reticulocyte system was approximately 5.5 h, suggesting it may possess moderate stability *in vivo* (Figure 1A). Polarity analysis based on amino acid composition revealed that the peptide contains both polar (acidic, basic, and uncharged) and nonpolar amino acids, exhibiting mixed polarity characteristics. Further analyses, including hydrophilicity profiling (Hopp and Woods method), hydropathy plot analysis across the sequence, and helical wheel projection, consistently demonstrated a strongly hydrophilic region in the central part of the peptide sequence, while the C-terminus displayed a pronounced hydrophobic tendency. This amphipathic distribution may influence its spatial conformation and intermolecular interactions (Figures 1B,C). The structure predicted by AlphaFold2, along with the pLDDT (predicted Local Distance Difference Test) scores, showed that the peptide adopts a typical α-helical conformation. All regions received pLDDT scores within the “Very high (>90)” range (indicated by dark blue), signifying a very high confidence level in the structural prediction (Figure 1D). Of note, the α-helical structure of this peptide has been experimentally validated by circular dichroism (CD) spectroscopy in our previous study (18), where the CD spectrum exhibited characteristic double minima at approximately 208 nm and 222 nm. The AlphaFold2 prediction is therefore consistent with the experimentally validated structure.

**Figure 1:**
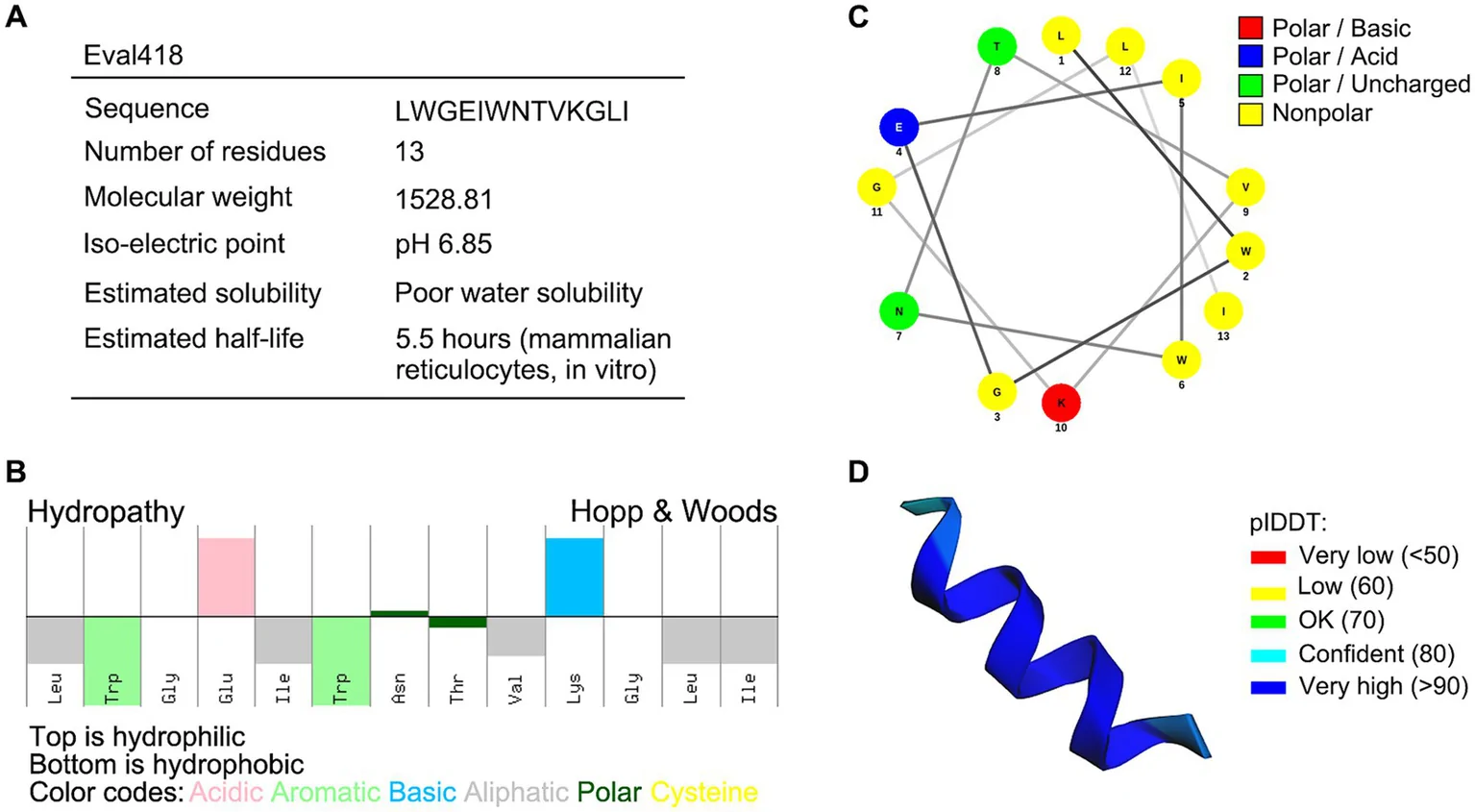
Comprehensive analysis of the physicochemical properties of the scorpion peptide Eval418 **(A)** basic parameters and sequence information of Eval418 **(B)** Hopp & Woods hydropathy scale analysis of Eval418 **(C)** helical wheel projection of Eval418 **(D)** predicted tertiary structure of Eval418.

### 3.2 Eval418 inhibits DTMUV replication and exhibits antiviral activity against different DTMUV isolates

To investigate the antiviral activity of Eval418 against DTMUV, DF-1 cells were first treated with Eval418 (0, 1.25, 2.5, 5, 10, and 20 μM), with Ribavirin (3 μM) included as a positive control. The selection of this Ribavirin concentration was based on preliminary dose–response experiments (Supplementary Figure 1), which confirmed its effective antiviral activity in our experimental system. The cells were subsequently infected with DTMUV at an MOI of 0.1, followed by continued treatment with the same concentration of Eval418 or Ribavirin throughout the experiment, as illustrated in the schematic diagram (Figure 2A). We then analyzed changes in DTMUV levels using Western blot, RT-qPCR, and IFA. Using the mouse anti-DTMUV NS3 polyclonal antibody, whose specificity was validated in our previous study (Supplementary Figures 2, 3), Western blot results demonstrated that Eval418 inhibited the expression of the DTMUV NS3 protein in a concentration-dependent manner. At concentrations of 10 μM or higher, NS3 expression was almost completely abolished, showing an inhibitory effect comparable to that of Ribavirin (Figure 2B). Consistent with the Western blot results, RT-qPCR analysis revealed a significant dose-dependent decrease in intracellular DTMUV RNA levels, with viral RNA becoming nearly undetectable at concentrations of 10–20 μM (Figure 2C). Furthermore, IFA was employed to visually assess the impact of Eval418 on DTMUV replication. The results confirmed a dose-dependent inhibitory effect of Eval418 on DTMUV. At concentrations of 10 μM and 20 μM, only minimal viral replication was detected in DF-1 cells (Figure 2D). These results collectively indicate that Eval418 potently inhibits DTMUV replication *in vitro*.

**Figure 2:**
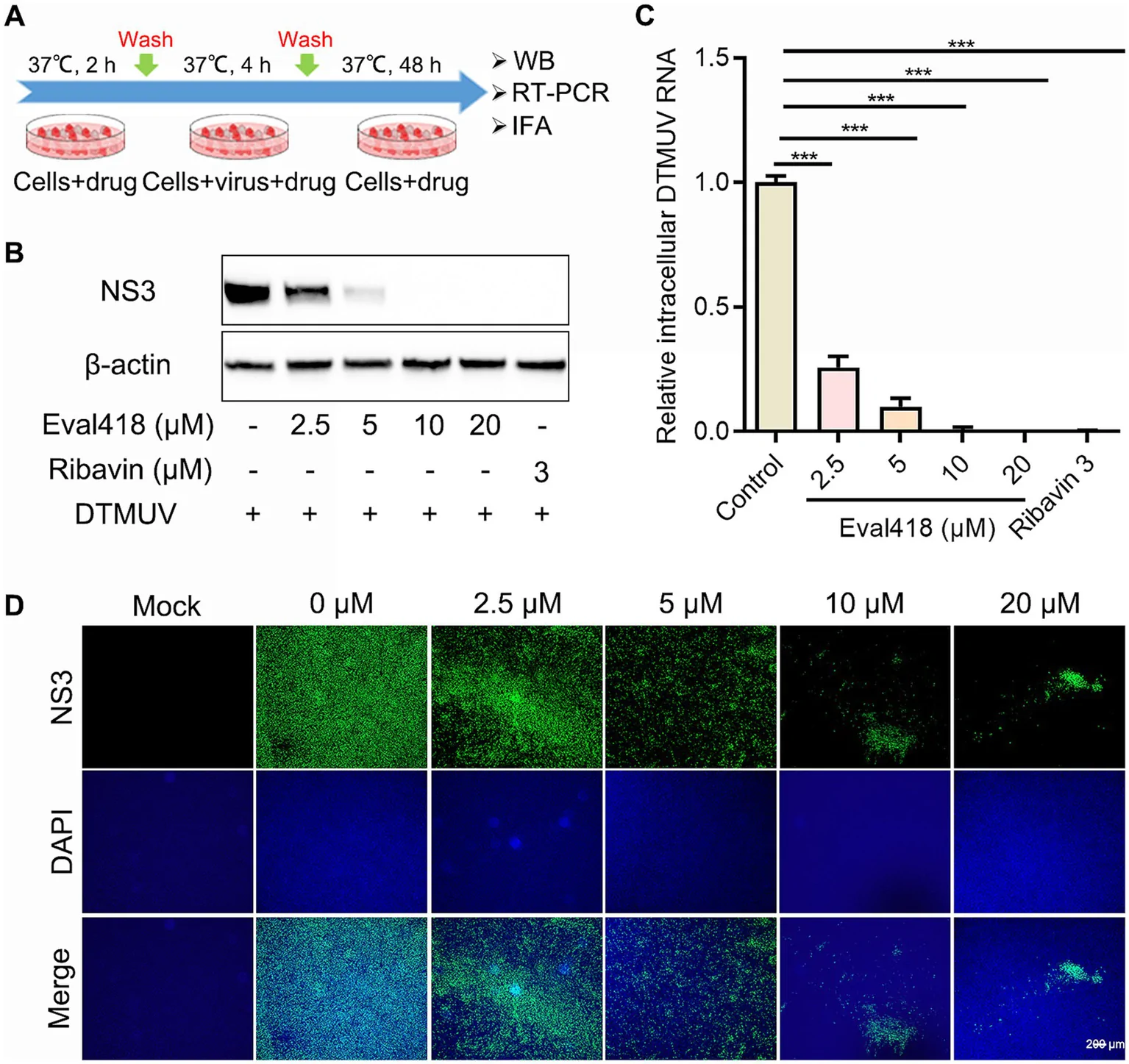
Eval418 potently inhibits DTMUV replication *in vitro*. **(A)** Schematic diagram of the experimental procedure: Cells were treated with Eval418, then co-incubated with DTMUV followed by another round of peptide treatment. Viral infection was finally assessed by Western blot, RT-qPCR, and IFA. **(B**–**D)** DF-1 cells were treated with Eval418 and infected with DTMUV according to the schematic in **(A)**. Eval418 concentrations were 0, 2.5, 5, 10, and 20 μM, and DTMUV was inoculated at an MOI of 0.1. Ribavirin (3 μM) served as the positive control. Western blot analysis of intracellular DTMUV NS3 protein expression levels using the mouse anti-DTMUV NS3 polyclonal antibody **(B)**. RT-qPCR analysis of relative intracellular DTMUV RNA levels. Data are presented as the mean ± SD from three independent experiments (*n* = 3). Statistical significance was determined using an unpaired two-tailed Student’s *t*-test. *** *p* < 0.001 **(C)**. IFA detection of intracellular DTMUV NS3 protein expression (green). Nuclei were stained with DAPI (blue). Merge shows the fluorescence overlay. Mock represents the uninfected and untreated control group. Scale bar: 200 μm **(D)**.

To further evaluate whether the antiviral activity of Eval418 extends to genetically distinct DTMUV isolates, we selected a representative strains isolated during a different epidemic periods for comparative analysis. Phylogenetic analysis comfirmed that the DTMUV strain used in this study and the DTMUV-2015 isolate belonged to distinct evolutionary branches (Supplementary Figure 4A). Western blot analysis showed that Eval418 inhibited NS3 protein expression of both isolates in a concentration-dependent manner. Nearly complete inhibition was observed at concentrations of 5 μM or higher for both isolates (Supplementary Figure 4B). These results indicate that the antiviral activity of Eval418 is not restricted to a single DTMUV isolate, although broader validation with a larger panel of isolates is warranted.

### 3.3 Biosafety profile of Eval418 and the influence of duck serum on its antiviral activity

To evaluate the suitability of Eval418 as a candidate antiviral agent against DTMUV, we first assessed its cytotoxicity in DF-1 and DEF cells, as well as its hemolytic activity against chicken and duck red blood cells (RBCs). MTT assays showed that treatment with Eval418 at concentrations up to 120 μM caused no detectable cytotoxicity, with cell viability remaining above 95% across all tested concentrations (Figures 3A,B). Consistently, hemolysis assays demonstrated that Eval418 exhibited negligible hemolytic activity, with hemolysis remaining below 2% at concentrations up to 120 μM (Figures 3C,D). To determine whether duck serum influences the antiviral activity of Eval418, the peptide was incubated with duck serum for different durations prior to antiviral evaluation. Western blot analysis showed that, in the absence of serum, Eval418 almost completely suppressed DTMUV NS3 expression throughout the entire incubation period (0–24 h). In contrast, pre-incubation with duck serum progressively reduced the antiviral activity of Eval418 in a time-dependent manner, with a marked loss of inhibition after 8 h of serum exposure. Notably, the serum solvent control group (serum + DMSO without peptide) showed no significant effect on NS3 expression at any time point, confirming that the serum and DMSO concentrations used in this assay did not interfere with DTMUV infection or replication (Figure 3E). These findings indicate that prolonged exposure to duck serum markedly compromises the antiviral activity of Eval418. It should be noted that the observed reduction in antiviral activity after serum incubation may result from multiple factors, including proteolytic degradation, non-specific binding to serum proteins, or peptide aggregation, rather than solely reflecting peptide degradation. Collectively, these results demonstrate that Eval418 exhibits favorable *in vitro* biocompatibility in the cell models tested, while its antiviral activity is progressively diminished following prolonged incubation with duck serum. However, it should be noted that these findings are limited to *in vitro* cell-based assays, and further *in vivo* studies are required to evaluate its systemic safety and pharmacokinetic profile.

**Figure 3:**
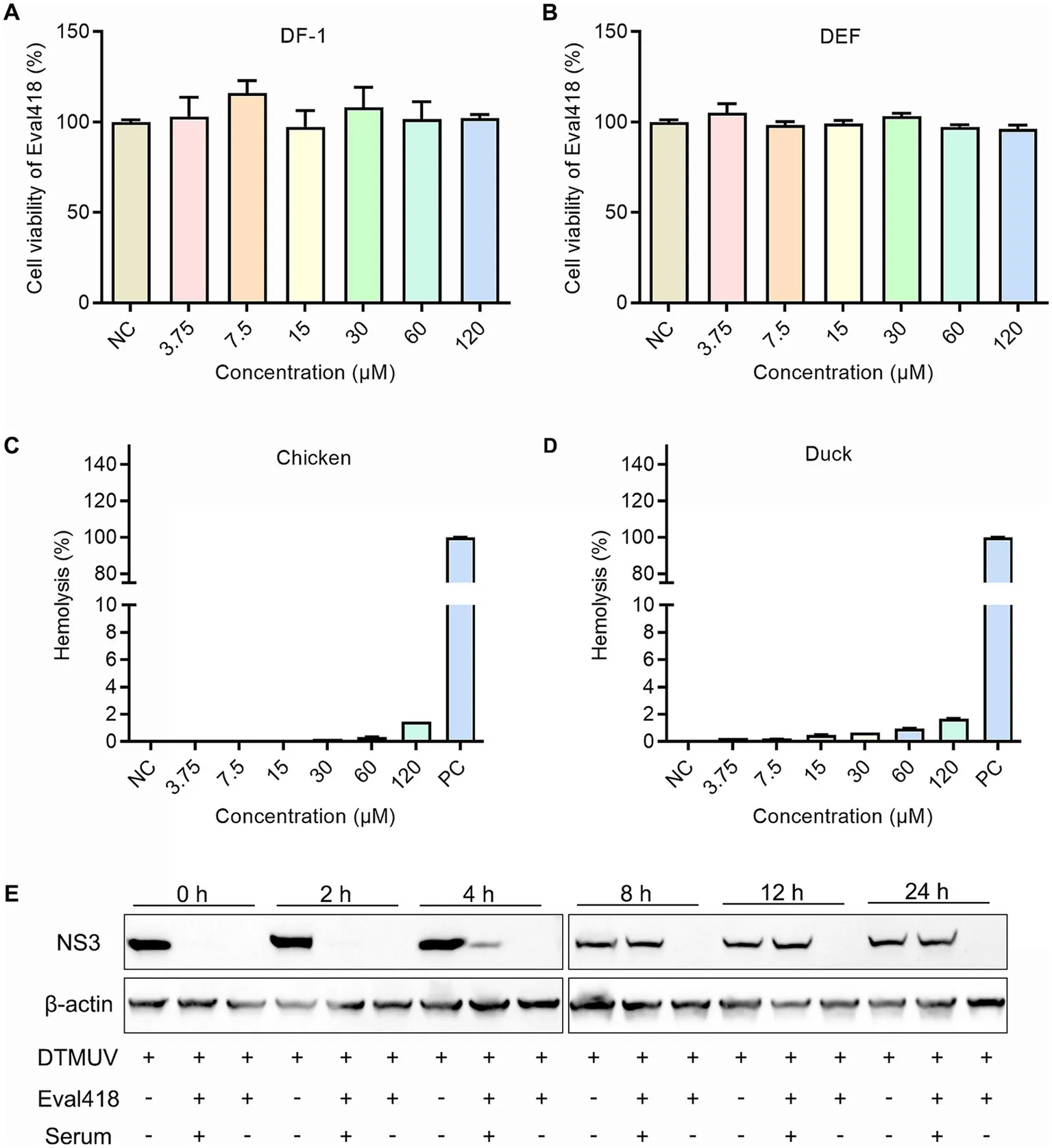
Biosafety profile of Eval418 and the influence of duck serum on its antiviral activity. **(A**,**B)** Cytotoxicity of the Eval418 peptide was determined by MTT assay. The concentration range was 0–120 μM. The half-maximal cytotoxic concentration (CC_50_) of Eval418 for DF-1 **(A)** and DEF **(B)** cells were greater than 120 μM. **(C**,**D)** Hemolytic activity of Eval418 in chicken **(C)** and duck **(D)** red blood cells were evaluated by hemolysis assay, also across a concentration range of 0–120 μM. The hemolysis rate of chicken and duck red blood cells was below 2% even at an Eval418 concentration as high as 120 μM. NC, Negative control; PC, Positive control. **(E)** Effect of duck serum incubation on the antiviral activity of Eval418. Eval418 was pre-incubated with 100% heat-inactivated duck serum or serum-free DMEM at 37 °C for the indicated times. After incubation, 10 μL of the peptide-serum mixture was added to the virus inoculum (final serum concentration: 1.8%; final Eval418 concentration: 20 μM), and the mixture was applied to DF-1 cells. Serum-free control and serum solvent control (DMSO) were included in parallel. Western blot analysis of intracellular DTMUV NS3 protein expression levels using the mouse anti-DTMUV NS3 polyclonal antibody.

### 3.4 Inhibitory effect of Eval418 on DTMUV replication at the coaddition stage

To determine the specific stage at which Eval418 inhibits DTMUV replication, we designed three distinct treatment regimens in DF-1 cells: pretreatment (Figure 4A), coaddition (Figure 4D), and postinfection (Figure 4G), with Ribavirin included as a positive control. The experimental results demonstrated that Eval418 almost completely inhibited DTMUV infection of DF-1 cells during the coaddition stage, both at the RNA level and protein expression level, and Ribavirin exhibited little or no inhibitory activity during this stage (Figures 4E,F). In contrast, Eval418 failed to inhibit DTMUV replication during either the pretreatment or postinfection stages (Figures 4B,C,H,I). In contrast, Ribavirin significantly reduced viral RNA levels and NS3 protein expression when added after viral infection had been established (Figures 4H,I). These results indicate that the inhibitory effect of Eval418 on DTMUV is highly dependent on the timing of treatment, exhibiting significant efficacy only when the virus and the peptide are applied to the cells simultaneously (coaddition).

**Figure 4:**
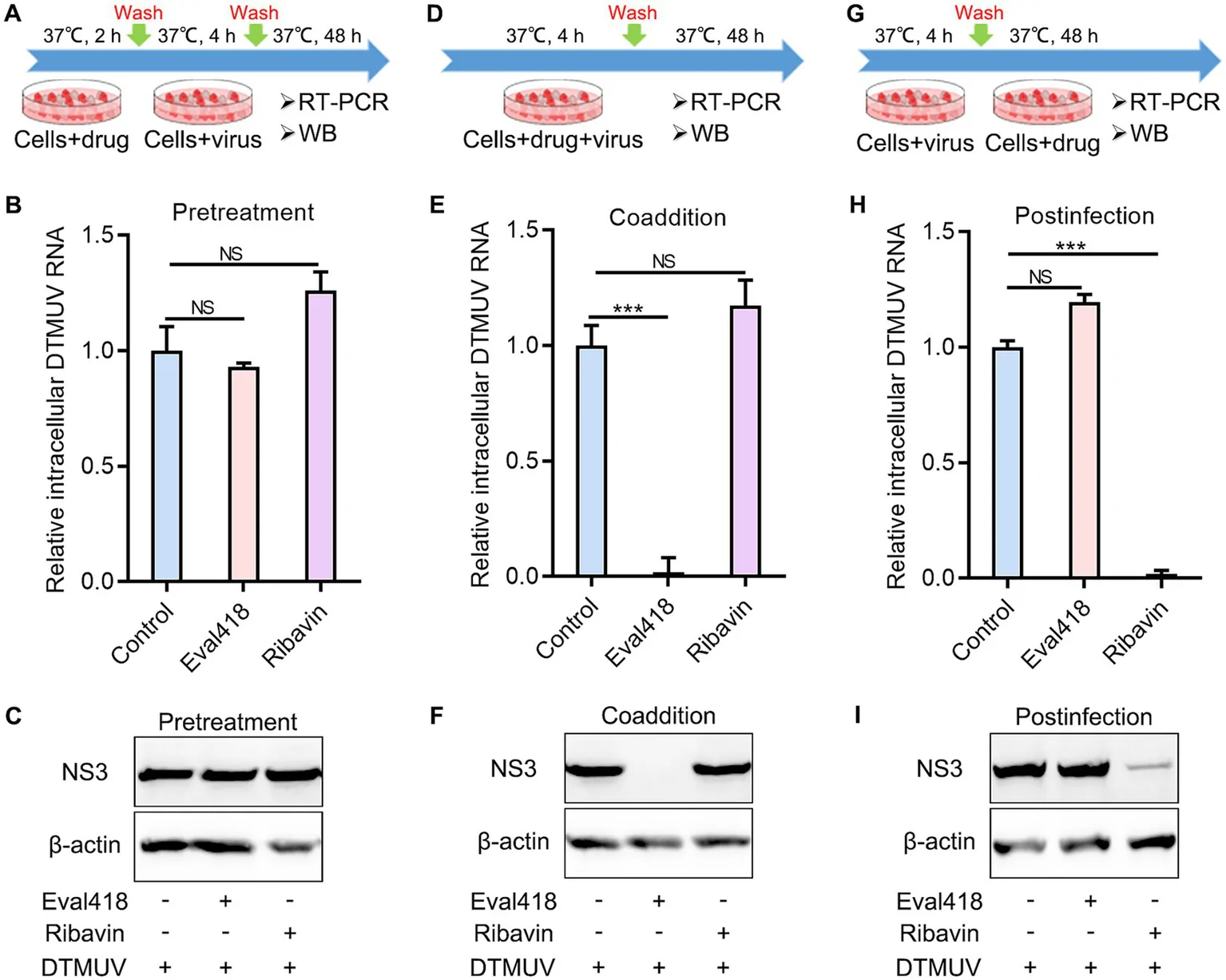
Stage-specific effect of Eval418 on DTMUV replication. **(A–C)** Pretreatment stage: DF-1 cells were pretreated with Eval418 (10 μM) or Ribavirin (3 μM) before DTMUV (MOI = 1) infection **(A)**. RT-qPCR analysis of intracellular DTMUV RNA levels **(B)**. Western blot analysis of intracellular NS3 protein expression **(C)**. **(D–F)** Coaddition stage: Eval418 (10 μM) or Ribavirin (3 μM) was added simultaneously with DTMUV (MOI = 1) during viral inoculation **(D)**. RT-qPCR analysis of RNA levels **(E)**. Western blot analysis of NS3 protein expression **(F)**. **(G–I)** Postinfection stage: Eval418 (10 μM) or Ribavirin (3 μM) was added after viral adsorption **(G)**. RT-qPCR analysis of RNA levels **(H)**. Western blot analysis of NS3 protein expression **(I)**. Data are presented as the mean ± SD from three independent experiments (*n* = 3). Statistical significance was determined using an unpaired two-tailed Student’s *t*-test. ***p < 0.001, NS, not significant.

### 3.5 Inhibition of Eval418 at multiple early stages of the DTMUV life cycle

Since Eval418 exhibited significant inhibitory effects only during the coaddition stage, we further focused on the early stages of the DTMUV life cycle. These include free virion activity (Figure 5A), cellular attachment (Figures 5D,K), and entry/fusion (Figure 5G), to investigate the mechanism of action of Eval418. The results showed that during the free virion stage, intracellular DTMUV RNA levels in the Eval418-treated group were significantly lower than those in the control group (Figure 5B). Concurrently, Western blot results confirmed that Eval418 almost completely inhibited NS3 protein expression, indicating that Eval418 can directly inactivate free DTMUV virions (Figure 5C). Similarly, during the viral attachment and entry/fusion stages, comparable results were observed using RT-qPCR (Figures 5E,H) and Western blot (Figures 5F,I) analyses. To more directly assess the effect of Eval418 on viral attachment, we performed a 4 °C binding assay. As shown in Figure 5J, Eval418 treatment significantly reduced the level of cell-associated DTMUV RNA compared to the untreated control group, indicating that Eval418 directly inhibits virus binding to the host cell surface. This suggests that Eval418 can also block the attachment of DTMUV to host cells and inhibit viral entry and membrane fusion. In summary, Eval418 effectively inhibits viral replication and protein expression within host cells by targeting multiple key steps of DTMUV infection, including free virions, attachment, and entry/fusion.

**Figure 5:**
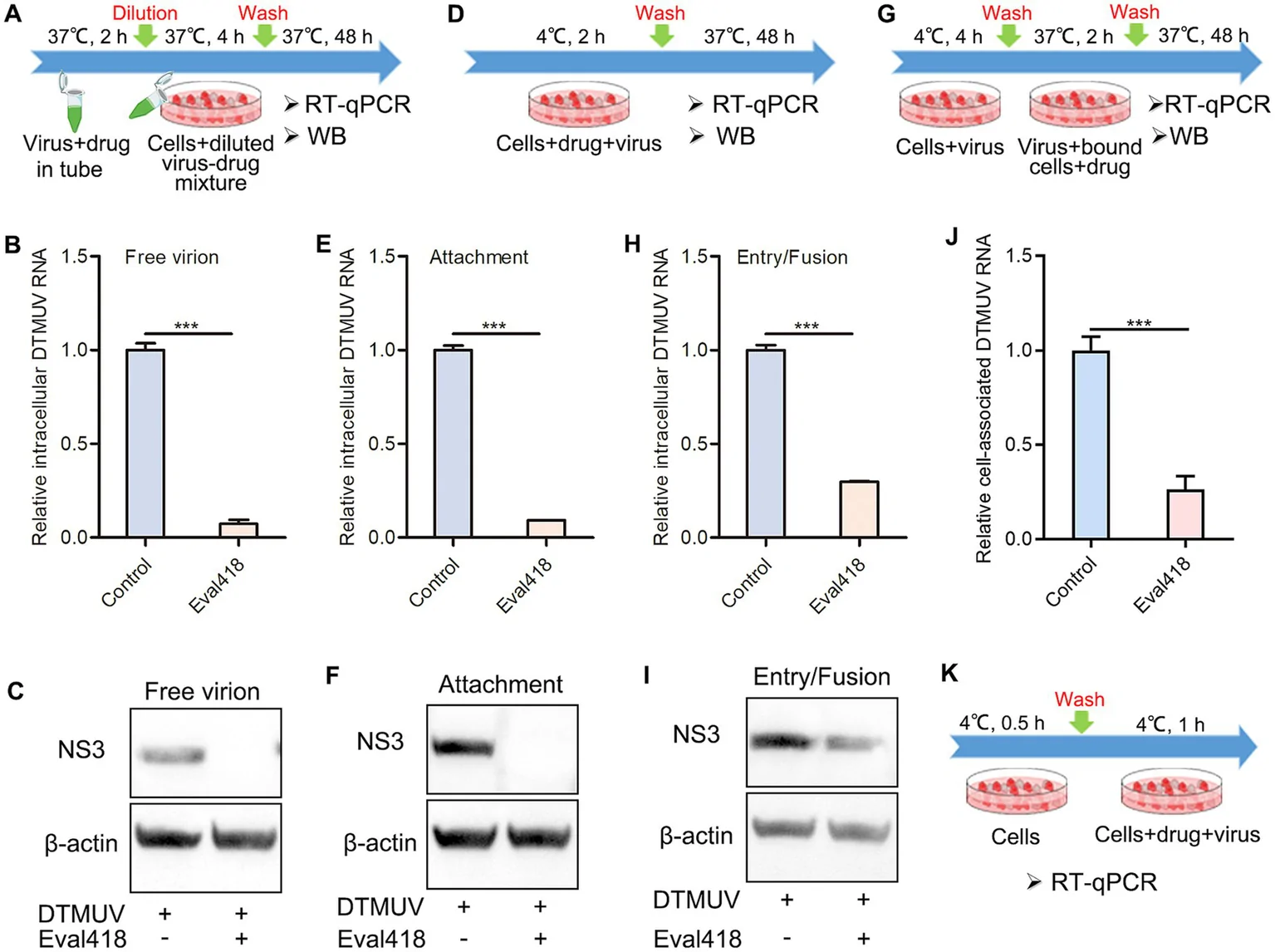
Inhibitory effect of Eval418 on key steps of DTMUV infection. **(A–C)** Free virion: Virus and peptide were treated according to the schematic diagram **(A)**, Eval418 concentration 0/10 μM, DTMUV inoculated at 1 MOI. RT-qPCR analysis of intracellular DTMUV RNA levels **(B)**. Western blot analysis of NS3 protein expression **(C)**. **(D–F)** Viral attachment: Cells, peptide, and virus were treated according to the schematic diagram **(D)**. RT-qPCR analysis of RNA levels **(E)**. Western blot analysis of NS3 protein expression **(F)**. **(G–I)** Viral entry/fusion: Cells and virus were treated followed by peptide addition according to the schematic diagram **(G)**. RT-qPCR analysis of RNA levels **(H)**. Western blot analysis of NS3 protein expression **(I)**. **(J)** DF-1 cells were incubated with DTMUV in the presence or absence of Eval418 at 4 °C for 1 h. After incubation, the cells were extensively washed with pre-cooled PBS to remove unbound viruses, and cell-associated DTMUV RNA was immediately extracted and quantified by RT-qPCR. **(K)** Schematic illustration of the viral attachment assay. Data are presented as the mean ± SD from three independent experiments (*n* = 3). Statistical significance was determined using an unpaired two-tailed Student’s *t*-test. ***p < 0.001.

### 3.6 Morphological damage of DTMUV virions induced by Eval418

Antiviral peptides can exert their effects through various mechanisms, which may involve inhibiting the viral replication cycle or directly inactivating viral particles. To further investigate the underlying mechanism by which Eval418 inhibits DTMUV infection and to confirm its ability to disrupt the integrity of DTMUV virions, we co-incubated Eval418 with DTMUV particles, performed negative staining, and subsequently observed the morphology of the virions using electron microscopy. We observed that in the DTMUV control group without Eval418 treatment, the virions were structurally intact, exhibiting the typical spherical morphology with distinct envelopes and full, well-defined particles (Figure 6A). In contrast, after treatment with Eval418, the DTMUV virions showed significant morphological disruption, appearing shrunken with collapsed or disrupted structures (Figure 6B). The TEM results demonstrate that Eval418 can physically damage the structural integrity of DTMUV particles, leading to a loss of infectivity.

**Figure 6:**
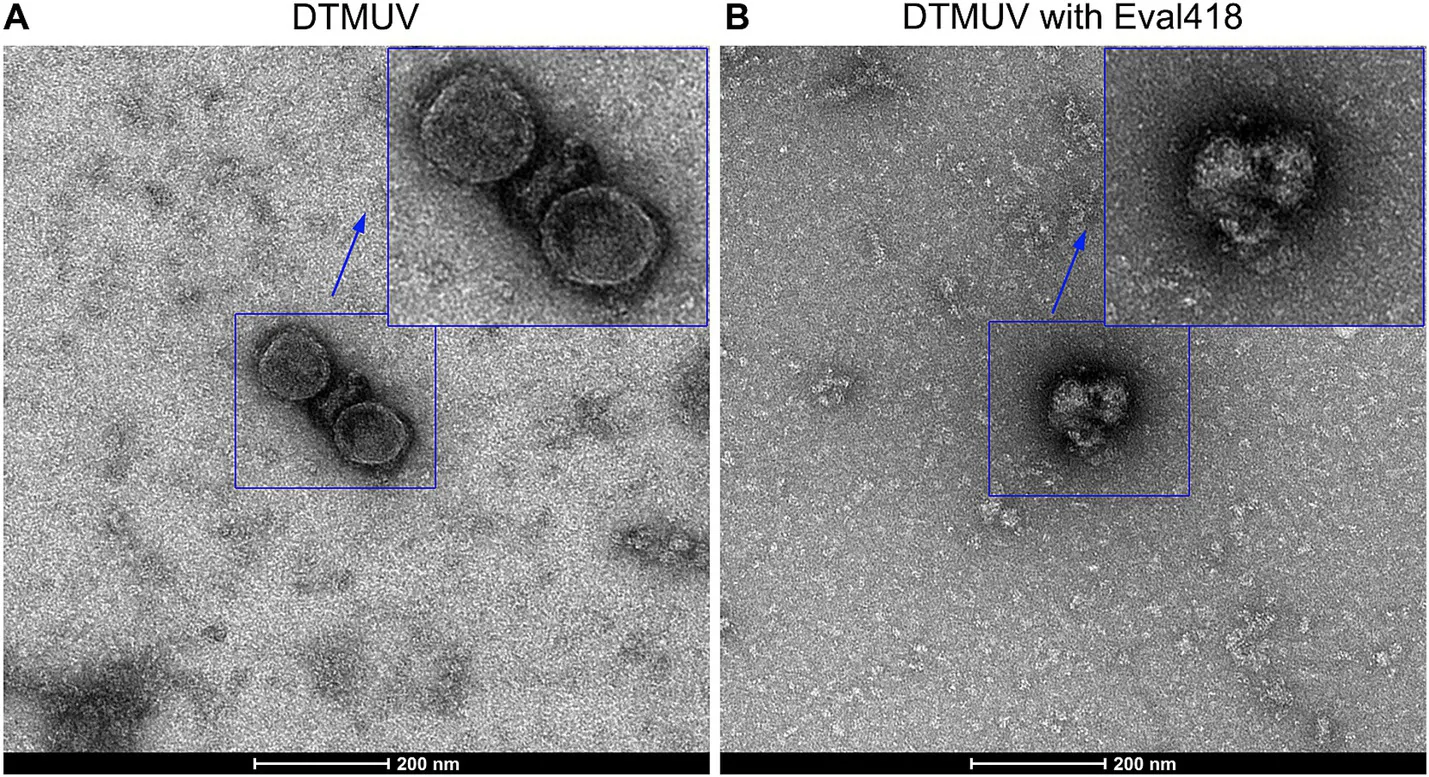
Eval418 induces morphological damage to DTMUV virions. **(A)** Untreated DTMUV virions. The untreated DTMUV virions exhibit an intact spherical structure with a distinct envelope and full, well-defined particles (the blue box indicates a locally enlarged area). **(B)** DTMUV virions treated with Eval418. The Eval418-treated DTMUV virions show severe morphological damage, appearing shrunken with structural collapse (the blue box indicates a locally enlarged area). Scale bar: 200 nm.

### 3.7 Molecular docking and molecular dynamics simulation of the NS3-Eval418 complex

The binding mode between NS3 and Eval418 was predicted via AlphaFold 3 protein-peptide docking. The results showed that the Eval418 peptide embedded into the binding pocket of NS3, forming a stable interface with complementary shape and charge (Figure 7A). Subsequently, a 100 ns molecular dynamics simulation was performed to comprehensively evaluate the dynamic stability of the complex. RMSD analysis revealed that the mean RMSD values for the protein backbone and the overall complex were 0.3651 nm and 0.4198 nm, respectively, and both converged after 21.7 ns; notably, the mean RMSD of the Eval418 peptide was only 0.2153 nm, considerably lower than that of the protein backbone, confirming its highly stable binding orientation (Figure 7B). RMSF analysis indicated that the flexible regions of NS3 were mainly distributed in the N-terminus and loop regions (e.g., residues 27–34, 112–123), whereas the core domains (e.g., residues 282–304, 487–499) exhibited RMSF values below 0.15 nm, providing a rigid scaffold for binding (Figure 7C). Throughout the simulation, the radius of gyration (Rg) remained stable within a narrow range of 2.3800 ± 0.0104 nm, indicating that the overall folded structure was preserved without any unfolding or expansion events (Figure 7D). The hydrogen-bond network at the NS3-Eval418 interface was monitored over the entire simulation, with an average of 6.71 interfacial hydrogen bonds maintained and never breaking during the 100 ns trajectory, which provided key non-covalent support for the sustained stability of the complex (Figure 7E). The solvent-accessible surface area of the complex remained stable throughout the simulation, with a total SASA of 310.37 ± 5.24 nm^2^, further confirming that the complex maintained a stable and well-folded conformation in solution (Figure 7F). The 3D Gibbs free energy landscape was constructed using RMSD and Rg as collective variables to further characterize the conformational stability of the NS3-Eval418 complex. The resulting free energy landscape (FEL) exhibited a single, narrow dominant energy basin with clearly distinguishable energy barriers, further confirming that the complex resides in a thermodynamically stable single conformational state (Figure 7G). Collectively, the 100 ns MD simulation results demonstrate that Eval418 can form a structurally stable and high-affinity complex with NS3, providing strong dynamic evidence for its potential as a bioactive modulator.

**Figure 7:**
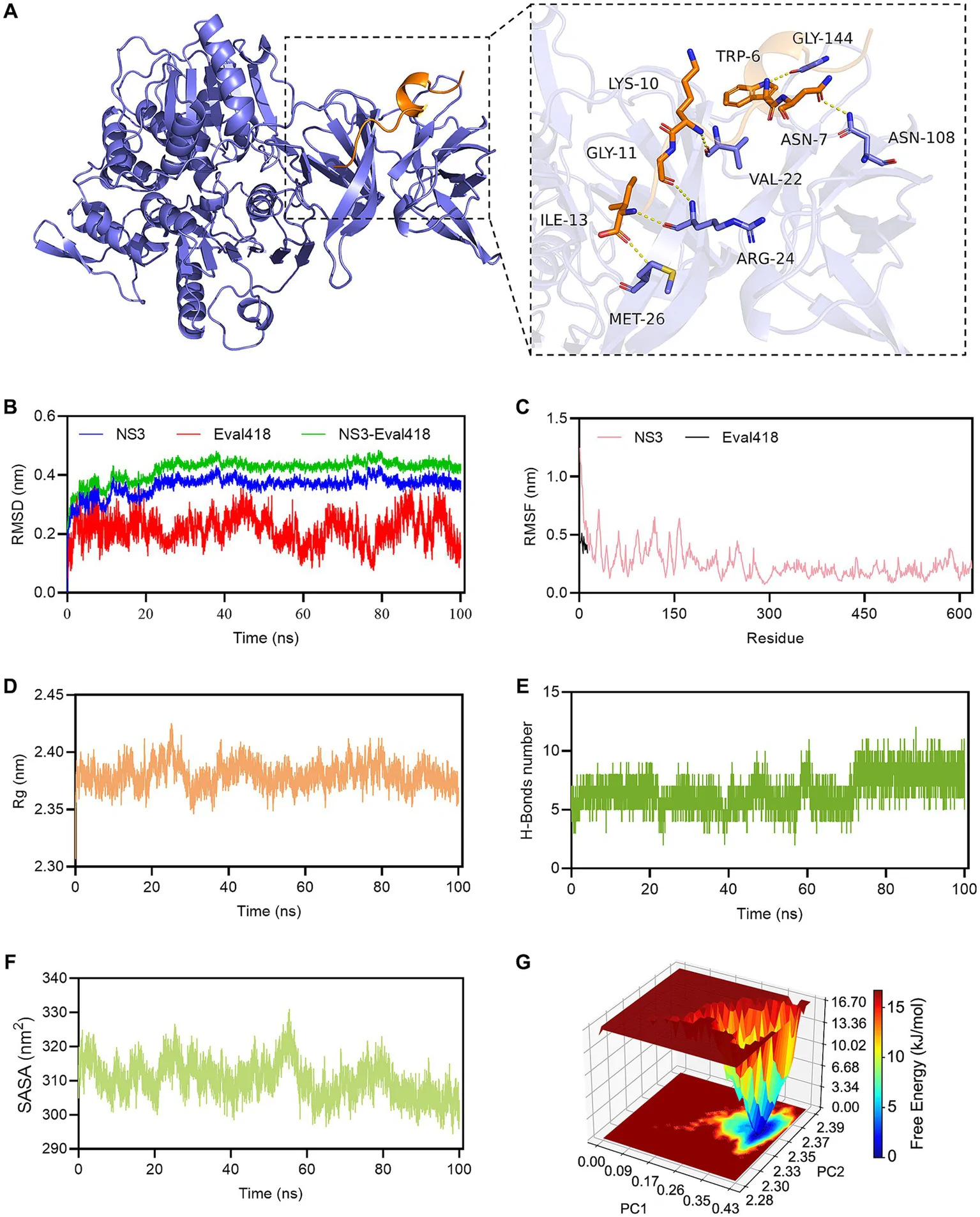
Molecular docking and molecular dynamics simulation of the NS3-Eval418 complex. **(A)** Molecular docking result showing the interaction between NS3 and the Eval418 peptide. The chemical structure of Eval418 (orange) is displayed in the binding pocket. **(B)** RMSD plots of NS3 (red), Eval418 (blue), and the NS3-Eval418 complex (green). **(C)** RMSF plot of the NS3-Eval418 complex. **(D)** Radius of gyration (*Rg*) plot of the NS3-Eval418 complex. **(E)** Number of hydrogen bonds at the NS3-Eval418 interface. **(F)** Solvent-accessible surface area (SASA) plot of the NS3-Eval418 complex. **(G)** 3D Gibbs free energy landscape of the NS3-Eval418 complex.

## 4 Discussion

DTMUV poses a substantial threat to the duck industry. Its high mutation rate and broad host range not only compromise the protective efficacy of existing vaccines but also highlight the urgent need for effective antiviral therapeutics (19–21). Over the past decades, numerous antiviral peptides have been discovered or designed, most of which possess potential virucidal activities (22, 23). Scorpion-derived peptides, owing to their unique mechanisms of action and low cytotoxicity, have emerged as an important library of lead compounds for antiviral drug development (24). In this study, we found that the scorpion-derived peptide Eval418 exhibits potent inhibitory activity against DTMUV.

The antiviral activity of Eval418 against DTMUV is consistent with previous findings obtained against other viruses. Zeng et al. (18) demonstrated that Eval418 significantly inhibited herpes simplex virus type 1 (HSV-1) infection by directly inactivating viral particles and blocking viral attachment; however, its antiviral activity against DTMUV had not previously been investigated. Here, we showed that Eval418 inhibited DTMUV replication in a dose-dependent manner by significantly reducing viral RNA levels and NS3 protein expression. At concentrations of 10 μM or above, viral replication was almost completely suppressed. Importantly, Ribavirin was included as a positive antiviral control in the present study, and Eval418 exhibited antiviral efficacy comparable to that of Ribavirin under the tested conditions (Figure 2). Notably, this peptide exhibited no significant cytotoxicity or hemolytic activity against DF-1 and DEF cells or chicken and duck red blood cells even at concentrations as high as 120 μM (Figure 3). Collectively, these findings indicate that Eval418 possesses potent antiviral activity.

The viral life cycle encompasses key stages such as attachment, entry, replication, and release. Most current antiviral drugs are designed to target these steps, aiming to terminate infection by selectively inhibiting one or multiple stages. For example, EGCG (Epigallocatechin-3-gallate) has been shown to exert multi-target antiviral effects during viral entry and immune modulation by directly disrupting viral particles, blocking the adsorption of DTMUV to host cells, and upregulating the expression of type I interferons (25). Curcumin effectively inhibits DTMUV infection, and its antiviral activity against DTMUV may be mediated by interfering with viral endocytosis and entry (26). Similarly, the antiviral activity of lycorine against DTMUV is primarily manifested by blocking the stages of viral internalization and entry. By directly interacting with viral particles and inhibiting post-entry steps, it significantly reduces viral replication (27). Furthermore, studies on the wasp venom peptide Protopolybia-MP III against HSV-1 reveal that it not only inhibits multiple stages of infection, including viral attachment, entry/fusion, and post-entry, but also directly disrupts the integrity of viral particles, demonstrating a multifaceted and synergistic antiviral mechanism (22). These studies collectively illustrate that intervention strategies targeting different stages of the viral life cycle constitute an effective approach for developing potent antiviral drugs.

Consistent with these previous reports, our time-of-addition experiments demonstrated that Eval418 acts predominantly during the early stage of DTMUV infection. Pretreatment of cells with Eval418 showed little antiviral activity, whereas simultaneous incubation of the peptide with virus almost completely abolished viral infection. In contrast, Eval418 exhibited minimal inhibitory activity when added after viral entry had occurred, whereas Ribavirin remained effective during the post-infection stage (Figure 4). These findings indicate that Eval418 and Ribavirin inhibit DTMUV through distinct antiviral mechanisms. Further mechanistic analyses demonstrated that Eval418 directly inactivated free virions, blocked viral attachment, and inhibited viral entry (Figure 5). Transmission electron microscopy (TEM) results provided visual confirmation that Eval418 can directly disrupt the structural integrity of DTMUV virions, causing viral envelope shrinkage and morphological collapse (Figure 6). Additionally, a 100 ns MD simulation was conducted to analyze the dynamic characteristics of the NS3-Eval418 complex. The MD simulation results, particularly the analyses of RMSD, RMSF, Rg values, and hydrogen bonds, provided critical evidence for evaluating the stability of the NS3-Eval418 protein complex and the tertiary structure of the protein upon ligand binding, including the hydrophobicity of amino acid residues. Collectively, the molecular docking and molecular dynamics simulations confirmed that Eval418 is capable of forming a structurally stable and high-affinity complex with NS3, providing a solid molecular dynamics basis for further elucidation of its mechanism of action and subsequent functional validation (Figure 7). This offers direct morphological evidence supporting its mechanism of “directly inactivating free virions.” This mechanism of viral inactivation through physical disruption of the viral capsid or envelope differs markedly from many conventional drugs that target viral replication enzymes or cellular factors. Consequently, it may be less prone to inducing viral drug resistance, representing a unique advantage.

The amphipathic nature of Eval418, characterized by a hydrophilic central region and a hydrophobic C-terminus (Figures 1B,C), may contributes to its antiviral mechanism. The hydrophobic C-terminal residues (Ile12 and Leu13) are predicted to interact with the viral lipid envelope, whereas the hydrophilic region may facilitate interactions with the viral surface or aqueous environment. Combined with its α-helical structure confirmed by circular dichroism (18) and AlphaFold2 prediction (Figure 1D), these structural characteristics may promote membrane insertion and disruption, providing a structural basis for the direct virucidal activity observed in this study.

An important finding of the present study is that duck serum progressively reduced the antiviral activity of Eval418. Although the peptide retained partial inhibitory activity following short-term serum incubation, prolonged incubation (8–24 h) almost completely abolished its antiviral effect. The mechanism underlying this reduction in antiviral activity remains to be fully elucidated. While the loss of activity may be attributable to proteolytic degradation, nonspecific binding to serum proteins, or other serum-mediated interactions that reduce peptide availability, direct evidence for peptide degradation (e.g., via HPLC or LC–MS) was not obtained in the present study and warrants further investigation. These findings suggest that maintaining the biological activity of Eval418 under physiological conditions remains a challenge and should be considered during future therapeutic development.

Another important advance of this study is the validation of Eval418 against genetically distinct DTMUV isolates. Phylogenetic analysis confirmed that the two DTMUV isolates evaluated in this study belong to distinct evolutionary branches, and Eval418 effectively inhibited viral replication in both isolates in a concentration-dependent manner (Supplementary Figure 4). While these results demonstrate that the antiviral activity of Eval418 is not restricted to a single laboratory strain, the limited number of isolates tested precludes conclusions regarding its broader antiviral spectrum. Further evaluation using a larger panel of field isolates is warranted to determine the full range of its activity.

The reduction in antiviral activity following prolonged serum incubation also highlights the need to optimize the stability of Eval418 under physiological conditions. Strategies such as peptide cyclization, PEGylation, histidine-rich modification, liposomal encapsulation, and nanoparticle-based delivery systems have been shown to improve peptide stability and bioavailability, and therefore represent promising approaches for enhancing the therapeutic potential of Eval418 in poultry.

The envelope-disrupting mechanism of Eval418 also raises the possibility that this peptide may possess antiviral activity against other enveloped viruses, as reported for scorpion-derived peptides active against Dengue virus (15) and HCV (16, 17). However, whether Eval418 is active against other economically important avian enveloped viruses, such as avian influenza virus or Newcastle disease virus, remains unknown and should be investigated in future studies.

Although scorpion venom contains neurotoxic peptides, Eval418 is a short 13-amino acid peptide that differs fundamentally from classical long-chain neurotoxins targeting ion channels. Together with its negligible cytotoxicity and hemolytic activity observed *in vitro*, these findings support a favorable preliminary safety profile in the tested cell models. Nevertheless, it should be noted that these safety data are limited to *in vitro* assays and do not reflect systemic toxicity or bioavailability *in vivo*. Comprehensive toxicological evaluation, pharmacokinetic characterization, biodistribution studies, and efficacy evaluation in ducks remain necessary before practical application.

In summary, this study has identified a scorpion peptide, Eval418, which efficiently and with low toxicity inhibits DTMUV replication at the cellular level. Its mechanism involves multiple early-stage interventions, including direct disruption of virions, blockage of viral attachment, and inhibition of viral entry. These findings provide a promising lead compound and novel mechanistic insights for the development of antiviral agents against DTMUV and other potential flaviviruses.

## 5 Conclusion

This study demonstrates that the scorpion-derived peptide Eval418 exhibits potent antiviral activity against DTMUV *in vitro*. Eval418 inhibited DTMUV replication in a concentration-dependent manner while exhibiting negligible cytotoxicity and hemolytic activity in the tested cell models, indicating favorable *in vitro* biocompatibility within the experimental conditions tested. Further *in vivo* studies are required to assess its systemic safety and therapeutic potential. Mechanistically, Eval418 exerts its antiviral effect primarily during the early stages of infection through a multi-target approach: directly inactivating free virions, blocking viral attachment to host cells, and inhibiting viral entry/fusion. Transmission electron microscopy provides direct visual evidence that Eval418 disrupts the morphological integrity of DTMUV virions, causing structural collapse. Furthermore, molecular docking and molecular dynamics simulations revealed that Eval418 binds stably to the NS3 protein, forming a high-affinity complex with favorable dynamic properties. In addition, Eval418 exhibited antiviral efficacy comparable to that of Ribavirin under the tested conditions and effectively inhibited two genetically distinct DTMUV isolates, suggesting its potential against multiple DTMUV strains, although broader validation with a larger panel of isolates is needed. However, prolonged incubation with duck serum progressively reduced its antiviral activity, highlighting the need for further optimization of peptide stability and formulation. Future studies should focus on elucidating the mechanism underlying serum-mediated loss of antiviral activity, evaluating pharmacokinetic properties and antiviral efficacy *in vivo*, and optimizing peptide delivery strategies to facilitate practical application in poultry.

## Data Availability

The original contributions presented in the study are included in the article/Supplementary material, further inquiries can be directed to the corresponding authors.

## Footnotes

1
https://web.expasy.org/protparam/

2
https://pepcalc.com/

3
http://lbqp.unb.br/NetWheels/

4
https://alphafold.ebi.ac.uk/

5
https://alphafoldserver.com/

6
https://valdes-tresanco-ms.github.io/gmx_MMPBSA/
