# Supplementary material for: The scorpion peptide Eval418 inhibits duck Tembusu virus replication by disrupting viral structure and early-stage infection
Source: Front Vet Sci. 2026 Sep 21;13:1944118. doi: 10.3389/fvets.2026.1944118 (PMC13637515; doi:10.3389/fvets.2026.1944118)
Supplement: Supplementary file 1 [file Image_1.pdf]

Supplementary Figure

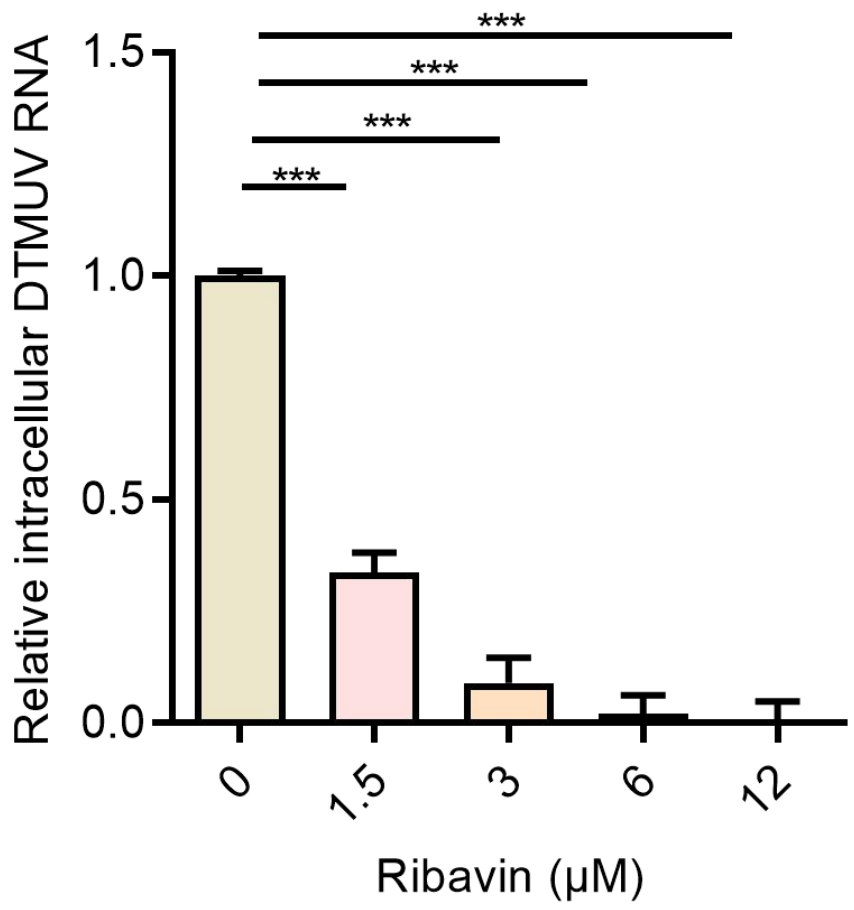

Supplementary Figure 1. Dose-response analysis of Ribavirin against DTMUV in DF-1 cells. Cells were infected with DTMUV (MOI=0.1) and treated with increasing concentrations of Ribavirin (0、1.5、3、6、12 μM). Intracellular DTMUV RNA levels were quantified by RT-qPCR at 48 h post-infection. Data are presented as the mean ± SD from three independent experiments (n = 3). Statistical significance was determined using an unpaired two-tailed Student's t-test. ***, P < 0.001.

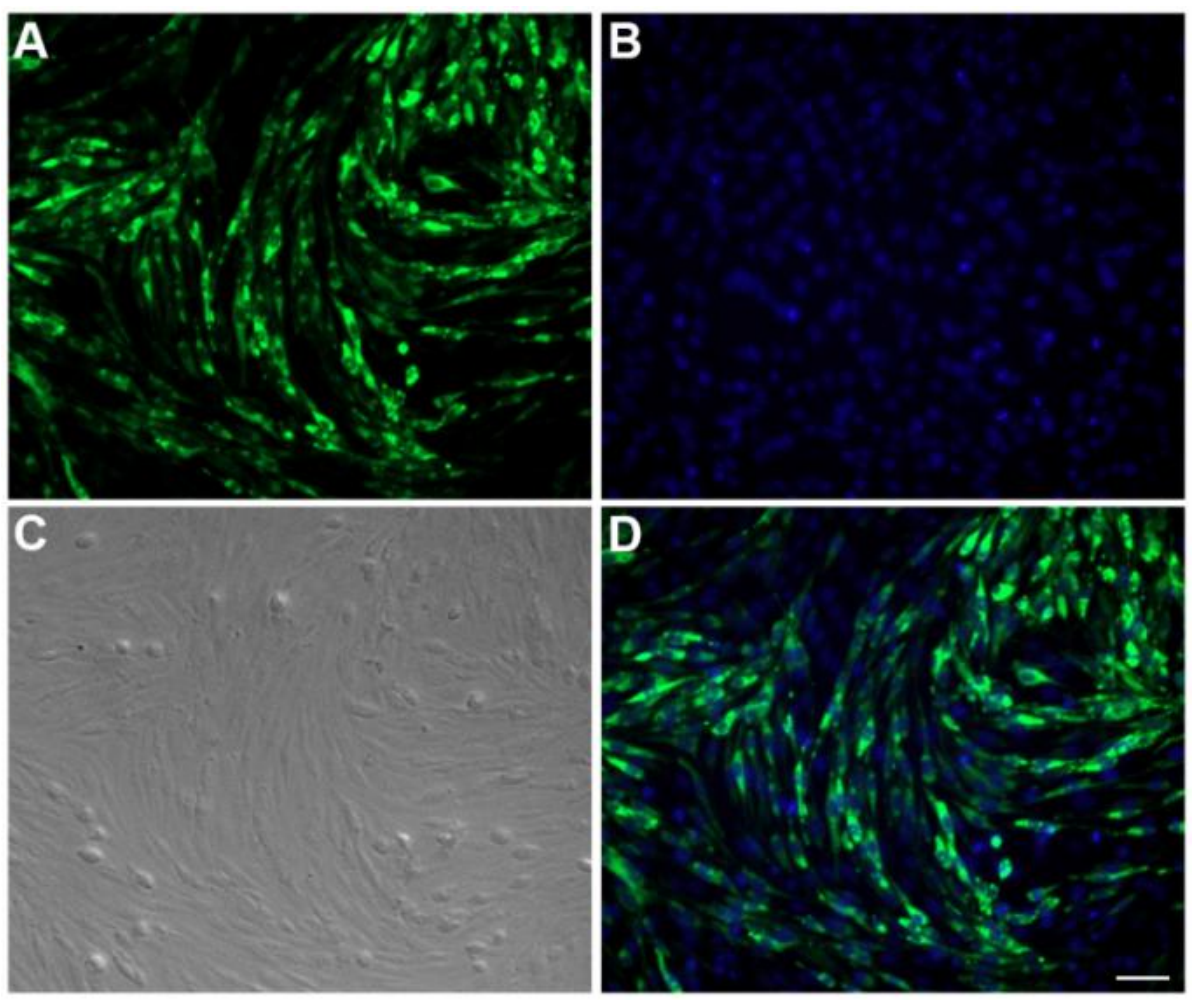

Supplementary Figure 2. Immunofluorescence assay (IFA) detection of the mouse polyclonal antibody. **(A)** Flag-NS3 protein (green); **(B)** DAPI (blue); **(C)** Bright field; **(D)** Merged image of fluorescence, DAPI, and bright field. Scale bar = 50 μm.

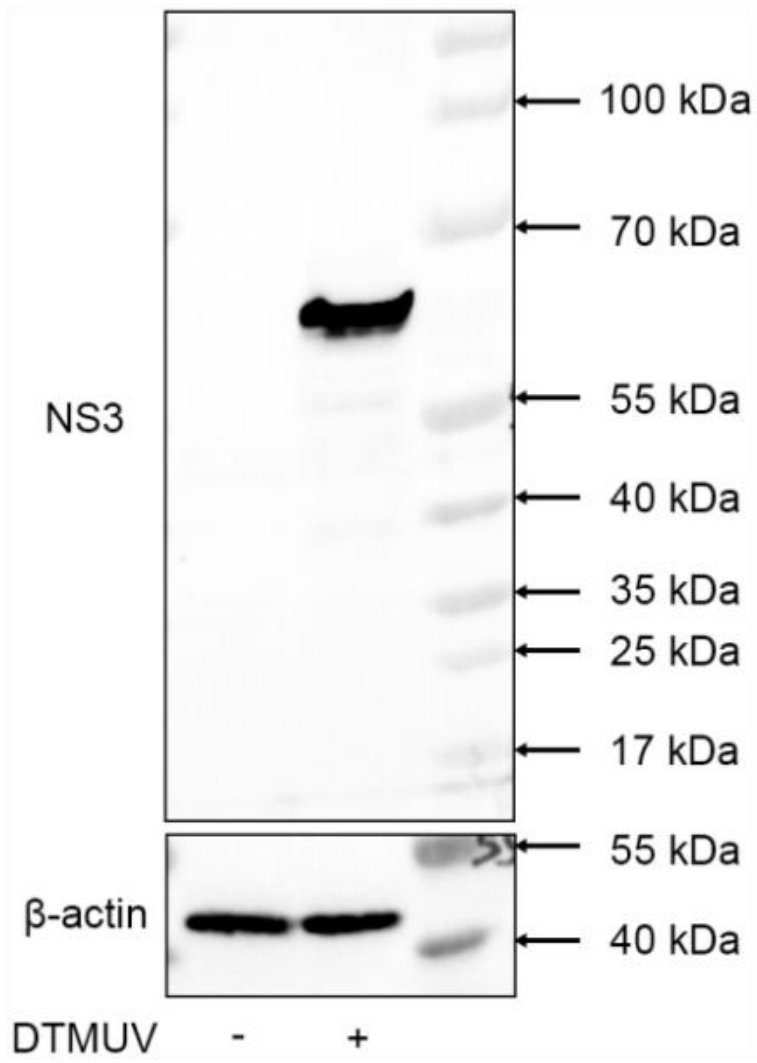

Supplementary Figure 3. Western blot (WB) detection of the mouse polyclonal antibody.

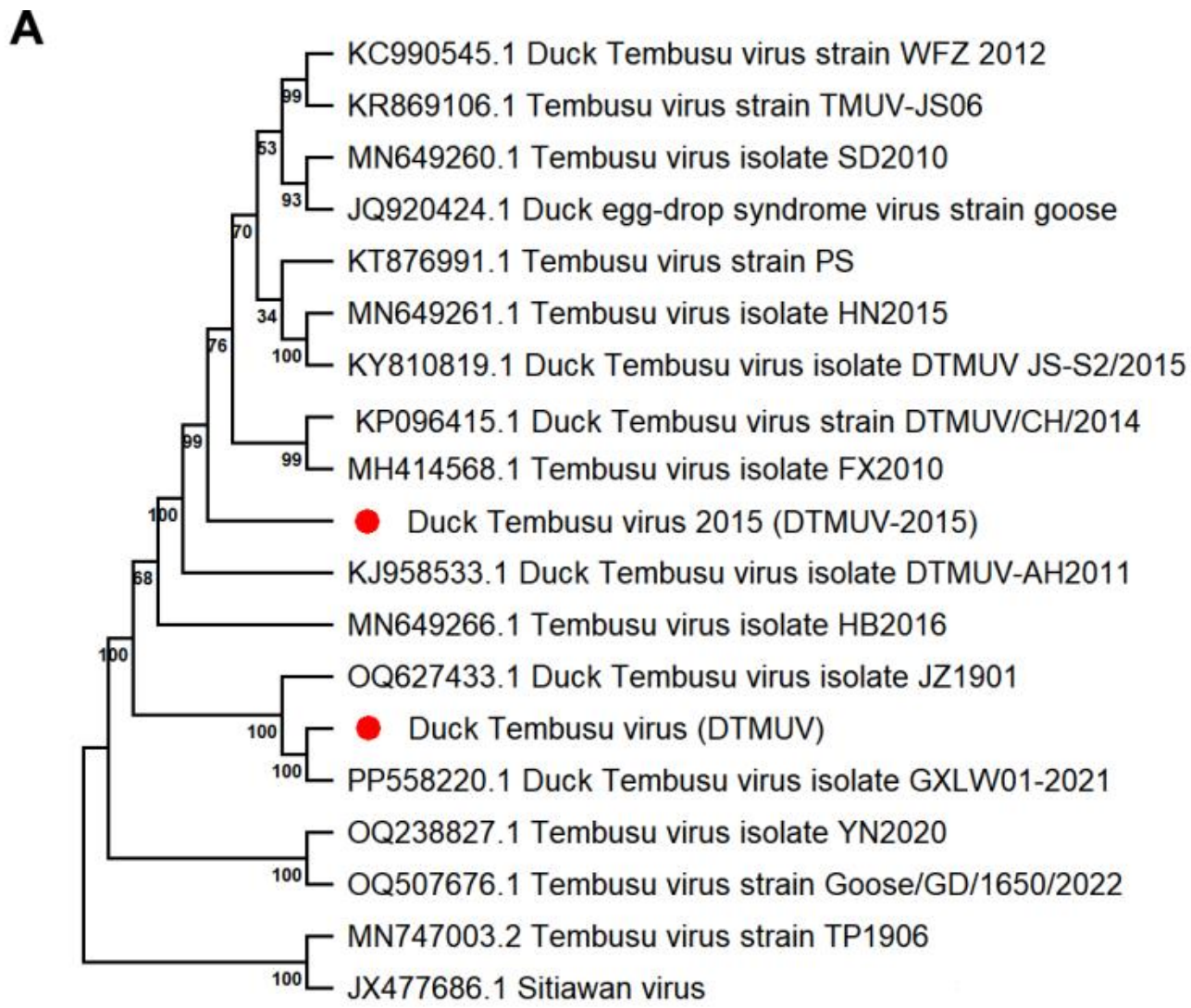

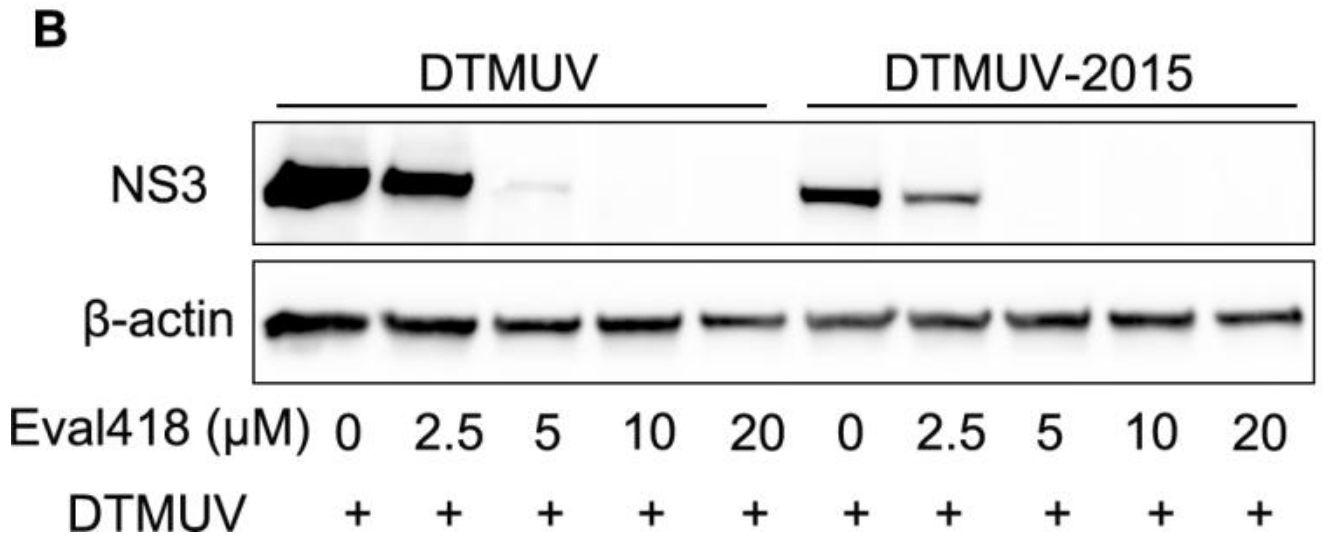

Supplementary Figure 4. Evaluation of Eval418 against genetically distinct DTMUV isolates. **(A)** Phylogenetic analysis of representative DTMUV isolates used in this study. The phylogenetic tree was constructed based on complete genome sequences, highlighting the DTMUV isolate used in this study and the DTMUV-2015 isolate selected for antiviral validation. **(B)** Western blot analysis of NS3 protein expression in DF-1 cells infected with either the DTMUV isolate or the DTMUV-2015 isolate following treatment with the indicated concentrations of Eval418.
