# Supplementary material for: The scorpion peptide Eval418 inhibits duck Tembusu virus replication by disrupting viral structure and early-stage infection
Source: Front Vet Sci. 2026 Sep 21;13:1944118. doi: 10.3389/fvets.2026.1944118 (PMC13637515; doi:10.3389/fvets.2026.1944118)

Full uncropped Gels and Blots image(s)

Figure 2-B

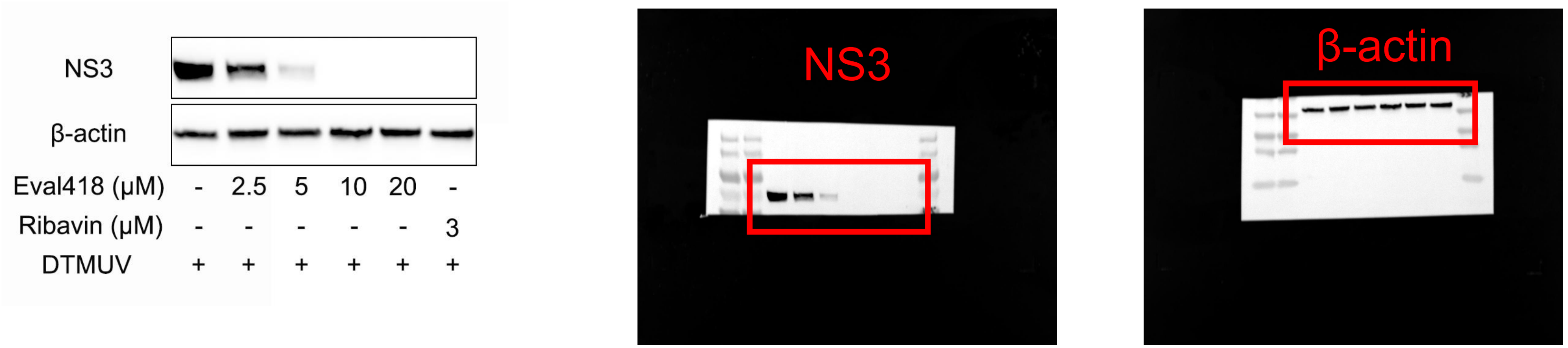

Figure 3-E

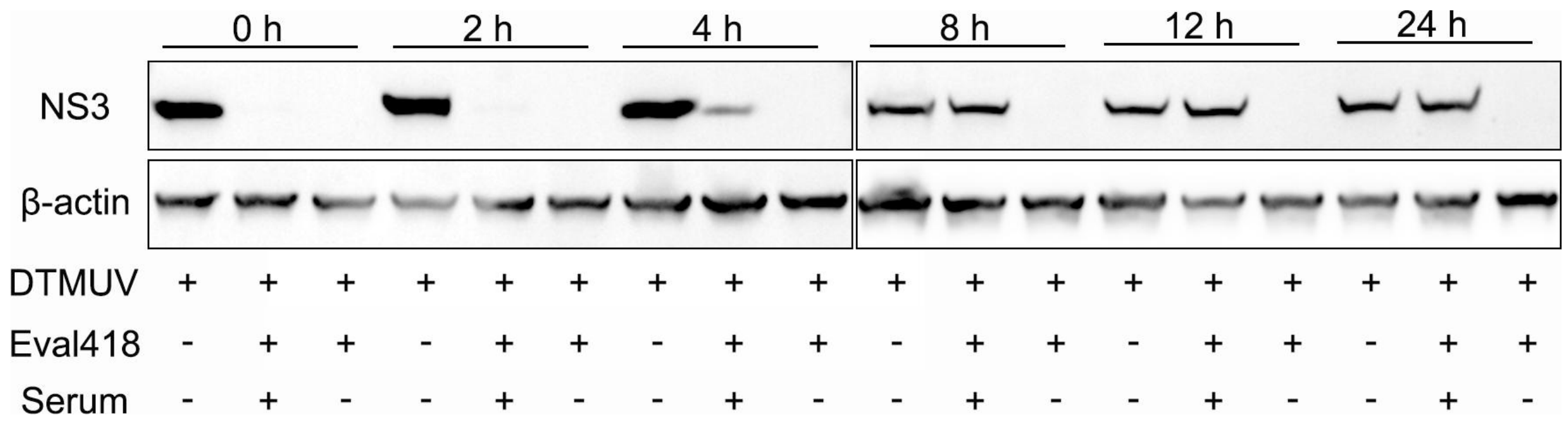
0 h
2 h
4 h
8 h
12 h
24 h
NS3
β-actin
DTMUV + + + + + + + + + + + + + + + + + +
Eval418 - + + - + + - + + - + + - + + - + +
Serum - + - - + - - + - - + - - + - - + -

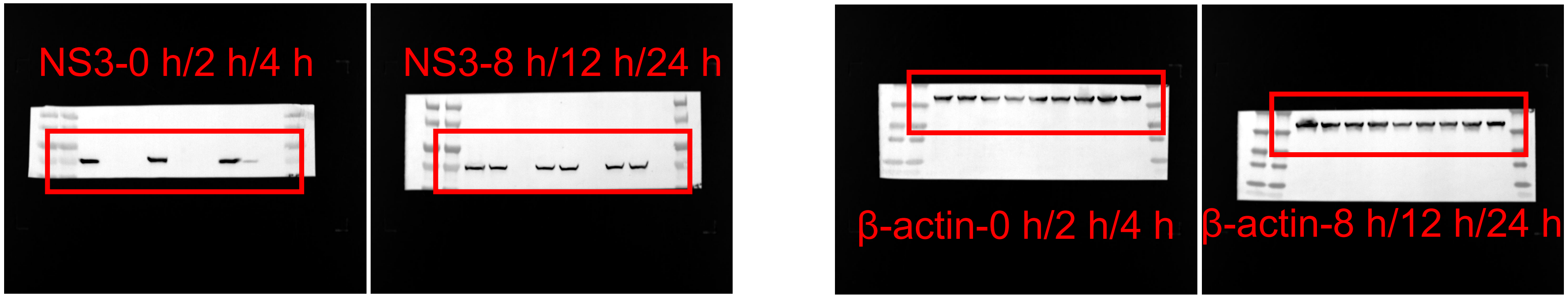
NS3-0 h/2 h/4 h
NS3-8 h/12 h/24 h
β-actin-0 h/2 h/4 h
β-actin-8 h/12 h/24 h

Figure 4-C, F, I

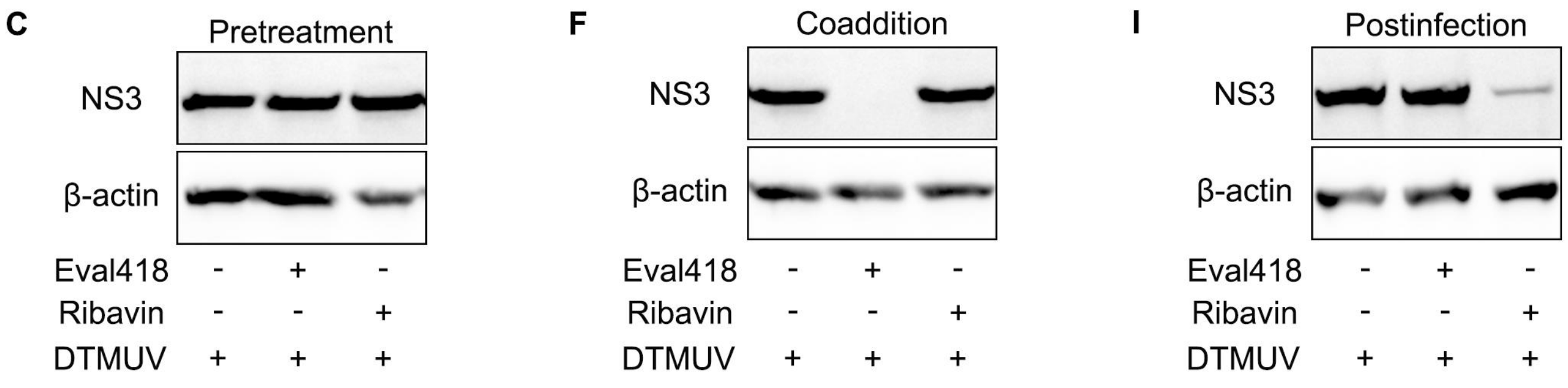

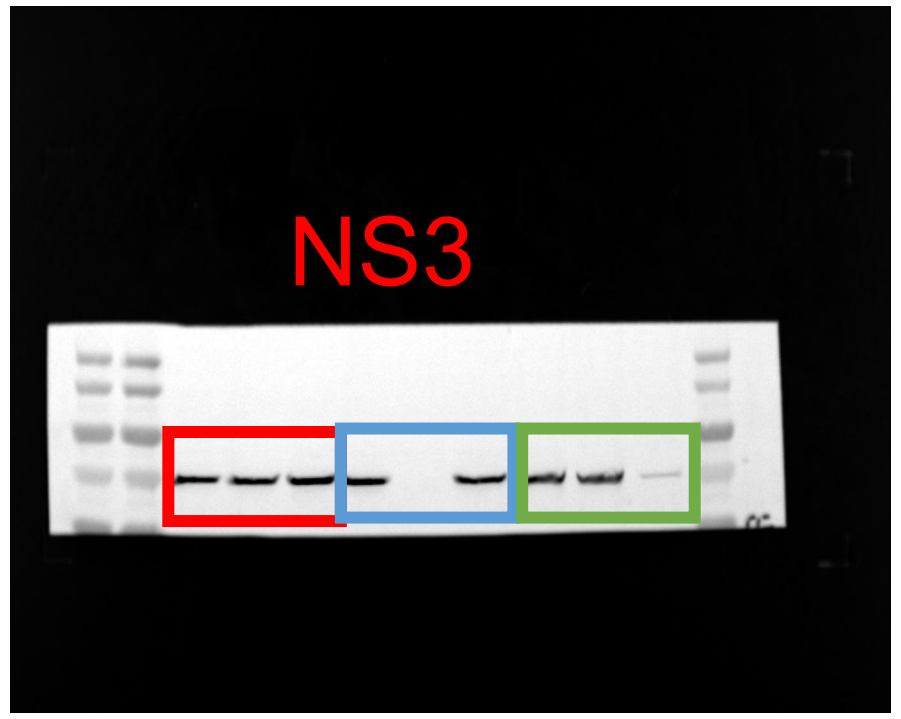

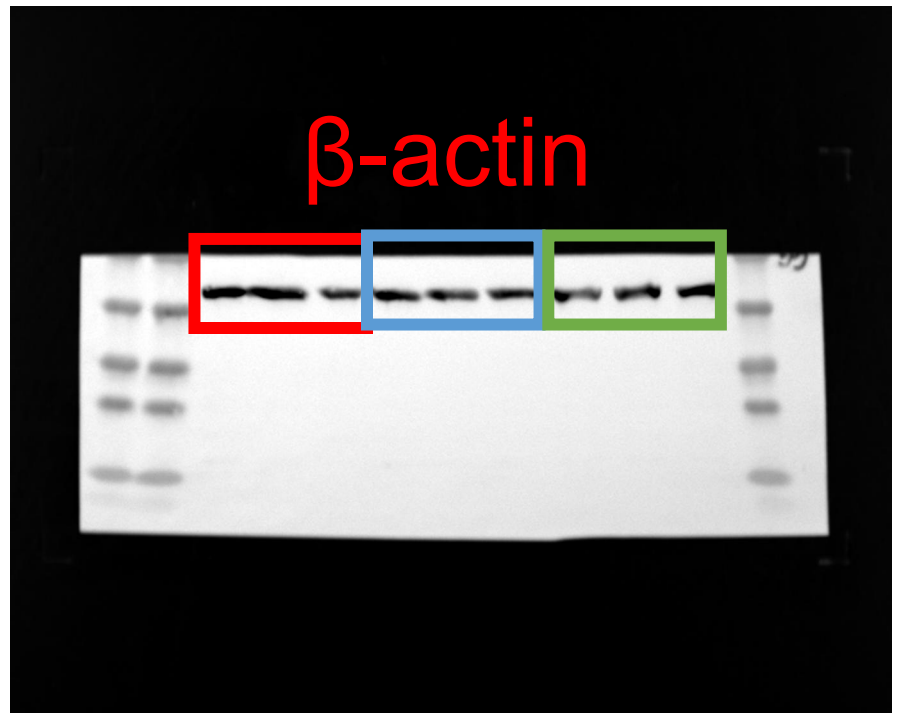

The red box represents the bands of Figure 4C for NS3 and β-actin.

The blue box represents the bands of Figure 4C for NS3 and β-actin.

The green box represents the bands of Figure 4C for NS3 and β-actin.

Figure 5-C, F, I

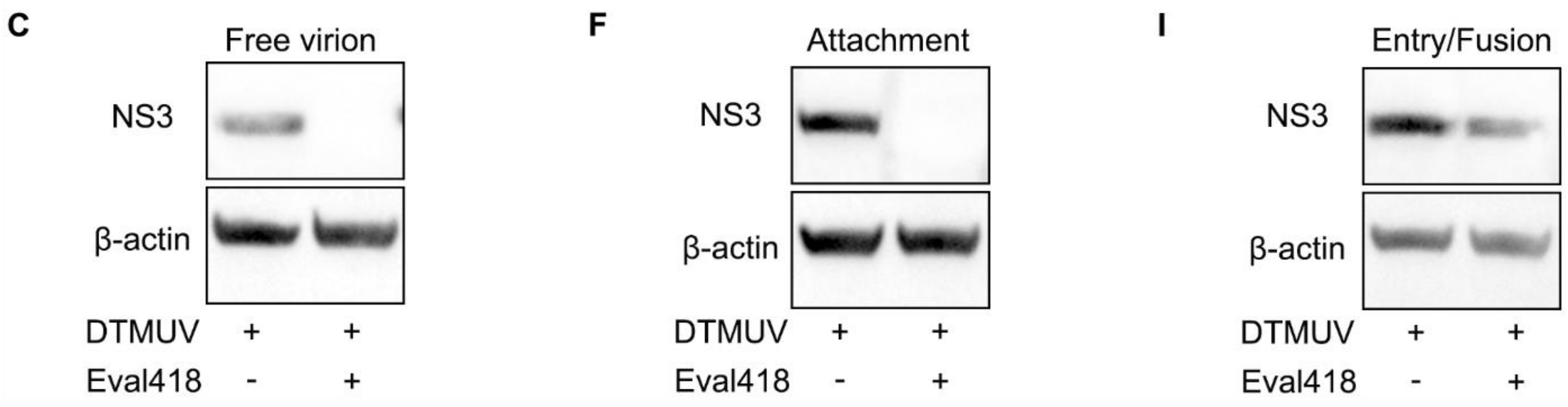

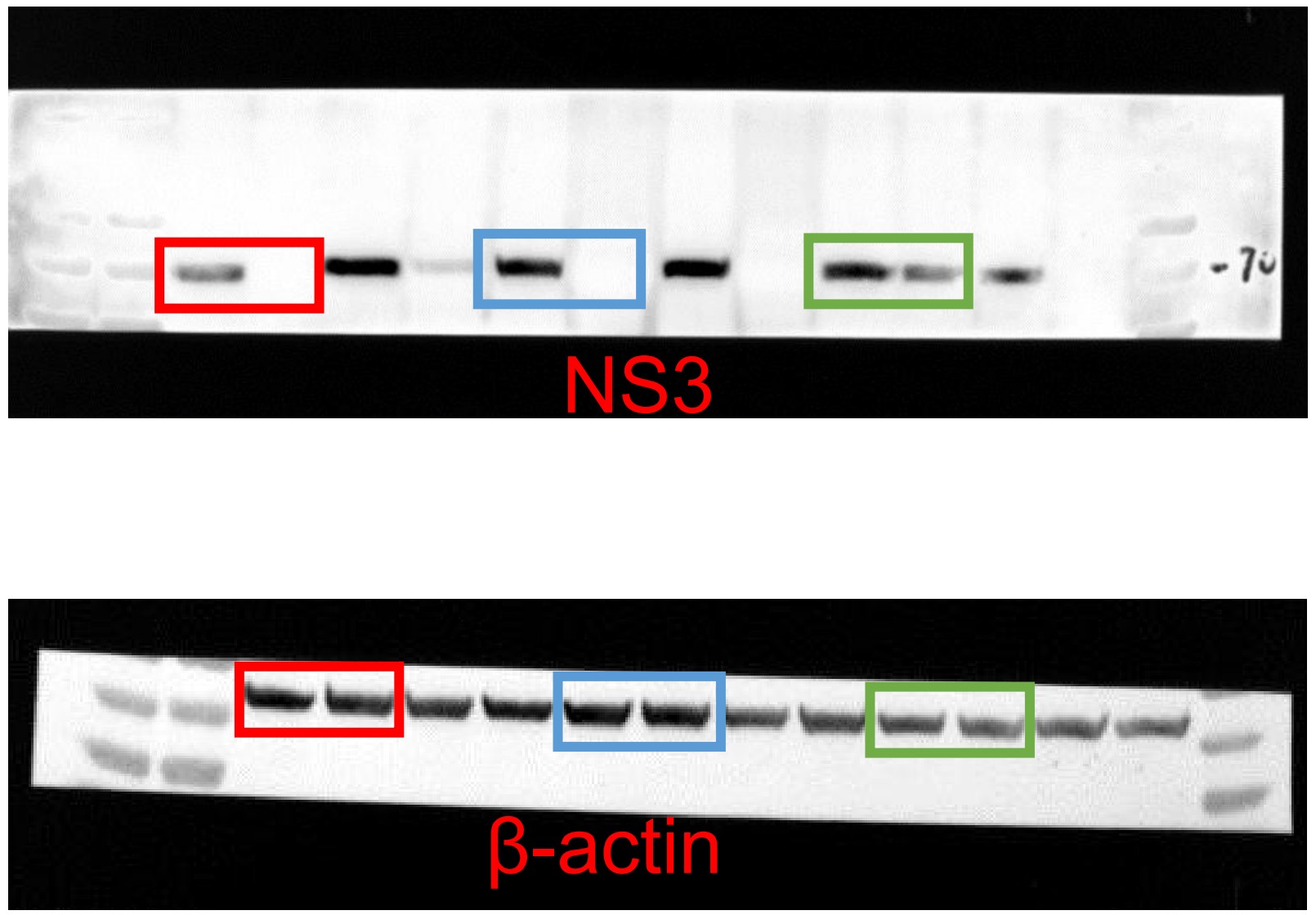

The red box represents the bands of Figure 5C for NS3 and β-actin.

The blue box represents the bands of Figure 5C for NS3 and β-actin.

The green box represents the bands of Figure 5C for NS3 and β-actin.

Supplementary Figure 4

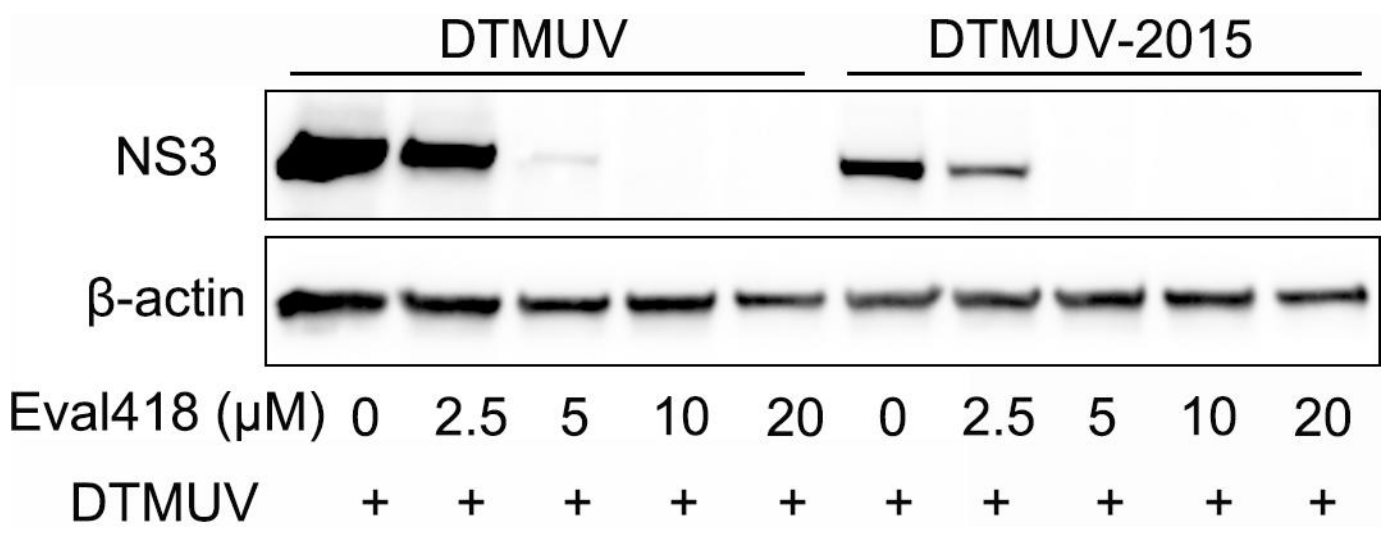

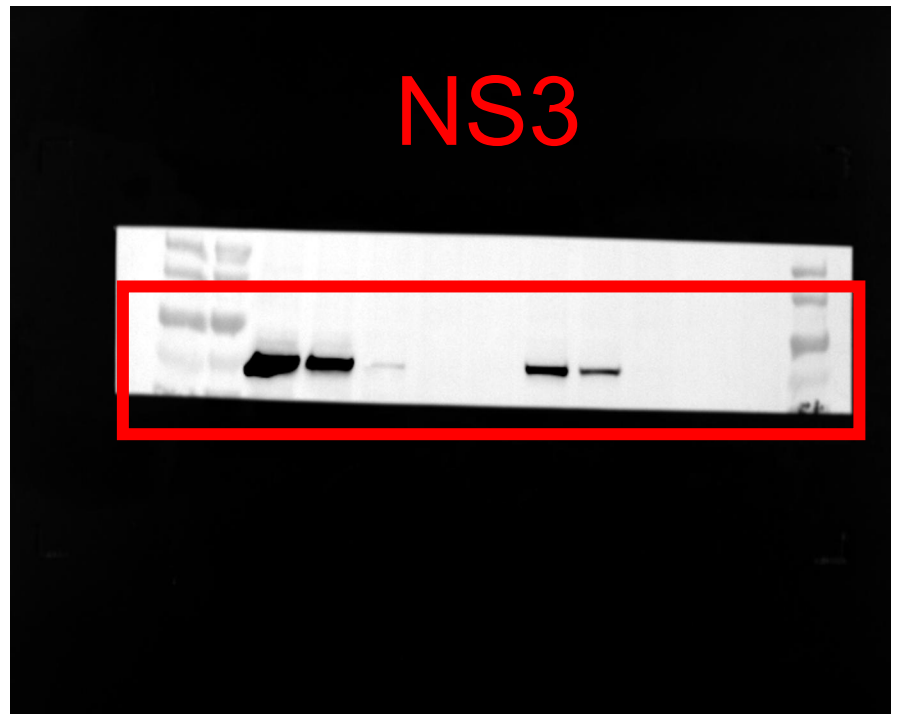

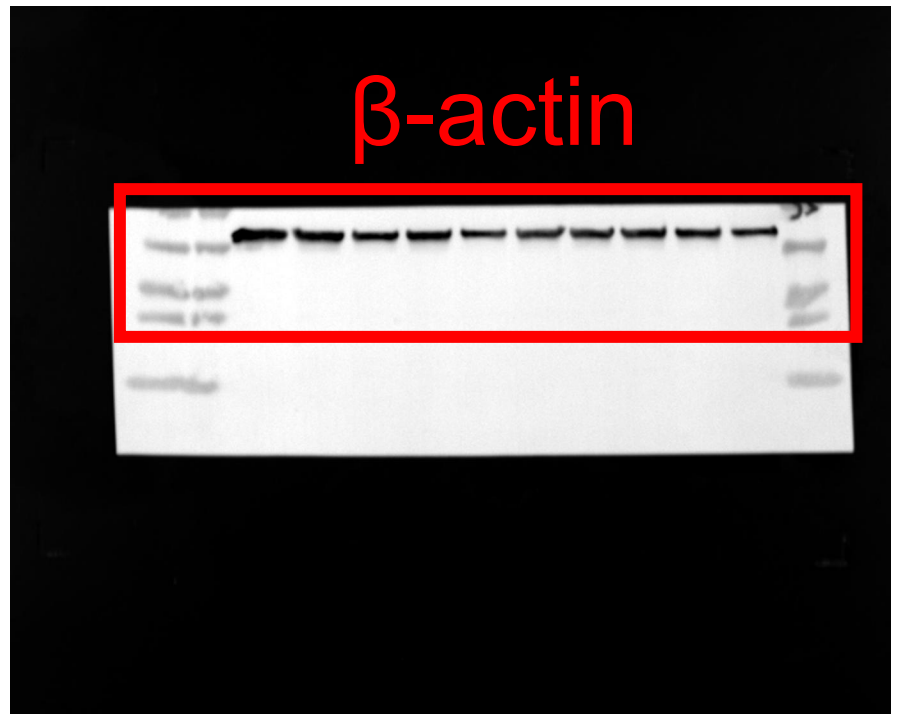

Supplement: Supplementary file 2 [file Supplementary_file_1.pdf]
